# RNA helicase A activity is inhibited by oncogenic transcription factor EWS-FLI1

**DOI:** 10.1093/nar/gku1328

**Published:** 2015-01-06

**Authors:** Hayriye Verda Erkizan, Jeffrey A. Schneider, Kamal Sajwan, Garrett T. Graham, Brittany Griffin, Sergey Chasovskikh, Sarah E. Youbi, Abraham Kallarakal, Maksymilian Chruszcz, Radhakrishnan Padmanabhan, John L. Casey, Aykut Üren, Jeffrey A. Toretsky

**Affiliations:** 1 Department of Oncology, Lombardi Comprehensive Cancer Center, Georgetown University, 3970 Reservoir Road NW, New Research Building E316, Washington, DC 20007, USA; 2 Department of Microbiology and Immunology, Georgetown University Medical Center, SW 309 Med-Dent, Washington, DC 20007, USA; 3 Department of Radiation Medicine, Georgetown University Medical Center, 3970 Reservoir Road NW, New Research Building E220, Washington, DC 20007, USA; 4 Department of Chemistry and Biochemistry, University of South Carolina, 631 Sumter Street, Columbia, SC 29208, USA

## Abstract

RNA helicases impact RNA structure and metabolism from transcription through translation, in part through protein interactions with transcription factors. However, there is limited knowledge on the role of transcription factor influence upon helicase activity. RNA helicase A (RHA) is a DExH-box RNA helicase that plays multiple roles in cellular biology, some functions requiring its activity as a helicase while others as a protein scaffold. The oncogenic transcription factor EWS-FLI1 requires RHA to enable Ewing sarcoma (ES) oncogenesis and growth; a small molecule, YK-4-279 disrupts this complex in cells. Our current study investigates the effect of EWS-FLI1 upon RHA helicase activity. We found that EWS-FLI1 reduces RHA helicase activity in a dose-dependent manner without affecting intrinsic ATPase activity; however, the RHA kinetics indicated a complex model. Using separated enantiomers, only (S)-YK-4-279 reverses the EWS-FLI1 inhibition of RHA helicase activity. We report a novel RNA binding property of EWS-FLI1 leading us to discover that YK-4-279 inhibition of RHA binding to EWS-FLI1 altered the RNA binding profile of both proteins. We conclude that EWS-FLI1 modulates RHA helicase activity causing changes in overall transcriptome processing. These findings could lead to both enhanced understanding of oncogenesis and provide targets for therapy.

## INTRODUCTION

Pathological changes in RNA processing are responsible for diseases ranging from spinal muscular atrophy (1) to cancer (2). RNA helicases are implicated in most cellular processes, including transcription, splicing, nuclear export, ribosome biogenesis and translation that are altered in pathology (3–6). RNA helicases have complex activities that often include dual enzymatic domains and multiple macromolecular scaffolds. Cancer, long recognized as a disease involving aberrant transcription, is rapidly becoming associated with the abnormal metabolism of RNA as a systematic pathogenetic mechanism (7). Thus, RNA helicases are being investigated as key proteins with multiple roles in oncogenesis that are likely context dependent (8). DDX3 is an example of one helicase that can function as an oncogene as reported in breast cancer (9) or as a tumor suppressor as demonstrated in hepatocellular carcinoma (10). There is a significant need both to understand the role of RNA helicases in oncogenesis and to create probes that will allow the study of the mechanisms of these key protein interactions.

Recent studies show that the complex interaction between RNA helicases and their protein partners often regulates helicase activity (11–14). Interacting protein partners can deregulate RNA helicase activity thus contributing to oncogenesis, even if the RNA helicases themselves are not mutated. For example, the modulation of eIF4A RNA helicase activity and specificity occurs through cofactor eIF4G which increases its adenosine triphosphatase (ATPase) activity (13) and RNA product release (12). Also, the DEAD-box protein Rok1 demonstrates increased RNA substrate specificity in the presence of its cofactor Rrp5 (15). More than one co-factor may exist for a given helicase, and these co-factors can have conditionally opposing effects on ATPase activity. In the case of Dbp5 (human homolog DDX19), the protein Gle1 along with hexakisphosphate is required for optimal enzymatic function in *Saccharomyces cerevisiae* (16–18). In an opposing mechanism, Dbp5 binding to Nup159 reduces the RNA affinity for Dbp5 (19). Despite the large body of work in yeast demonstrating a wide variety of cofactor modulated effects upon helicase activity, the role of oncogenes, as helicase cofactors, requires separate disease-specific evaluation as these protein cofactors determine the context-dependent roles of RNA helicases.

RNA helicase A (RHA, DHX9, a.k.a. NDHII) is a member of the DExH subgroup of RNA helicases; it was first purified and characterized as an RNA/RNA and RNA/DNA helicase with 3′ to 5′ directionality in HeLa cells (20). RHA is required for normal gastrulation as demonstrated by the RHA-null embryo, which does not survive beyond day E7.5 due to the apoptotic cell death of embryonic ectodermal cells (21). The *Drosophila* homolog MLE (*Maleless*) protein, which exhibits 51% identity at the amino acid level to human RHA, is part of the male-specific lethal (MSL) complex subunit required to increase the transcription of X-linked genes in drosophila males (22,23). RHA is a scaffolding protein for breast cancer 1 (BRCA1) that links BRCA1 to the RNA polymerase II holoenzyme (24). Cyclic adenosine monophosphate (AMP) response element-binding protein-binding protein (CBP) binds to RHA, modulating its activity and regulating RNA polymerase II (25,26). RHA also regulates the translation of JUND by binding the 5′-terminal post-transcriptional control element, which facilitates polyribosome association (27). A recent study identified RHA as a modifier of ABT-737 resistance in an Eμ-myc/Bcl-2 lymphoma mouse (28). RHA also shows activity in the maintenance of genomic stability by unwinding intra-molecular triplex (H-DNA), RNA containing R-loops and G-quadruplexes (29–31). These data support RHA as a putative key accessory protein in oncogenesis.

Our investigation of protein partners for the Ewing sarcoma (ES) oncoprotein EWS-FLI1, a protein product of the t(11;22)(q24;q12) (32), identified RHA as a critical partner for transformation (33). We identified a novel small molecule, YK-4-279, based on an empirical screen using full-length EWS-FLI1; (S)-YK-4-279 prevents RHA from binding to EWS-FLI1 in treated ES cells (34). In addition, EWS-FLI1 transcript activation assays and Cyclin D1 isomer switching also show that only the (S)-YK-4-279 is an active inhibitor (35). When RHA is blocked from binding to EWS-FLI1 cells die by apoptosis (34), yet when shRNA reduces EWS-FLI1 levels, ES cells undergo a senescent-like death (36,37). One interpretation is that a functional relationship between EWS-FLI1 and RHA exists such that their dissociation, in contrast to disappearance, would shift a balance from tumor growth to apoptosis.

EWS–FLI1 interaction with RHA occurs near the helicase domain (33), therefore, we tested the effect of EWS-FLI1 upon RHA enzymatic activity. We show that purified RHA directly binds to recombinant EWS-FLI1 leading to reduced RHA helicase activity. Since the inhibition of RHA helicase activity did not follow basic kinetic models, we utilized atomic force microscopy (AFM) to directly observe protein–RNA interactions. The AFM confirmed a novel finding that EWS-FLI1 binds directly to RNA, and provided a mechanistic explanation of the protein–protein interaction effect on RHA activity. Consistent with prior enantiospecific inhibition of complex formation, only (S)-YK-4-279 was able to reverse the EWS-FLI1 inhibition of RHA helicase activity. Finally, to confirm that EWS-FLI1 and RHA bind a common subset of RNA in cells, YK-4-279-treated ES cells showed a shift in the RNA immunoprecipitation (RIP) profile. Overall, these investigations inform the interaction of EWS-FLI1 and RHA *in vitro* and demonstrate a functional effect of a small molecule protein–protein interaction inhibitor upon ES cells.

## MATERIALS AND METHODS

### Chemicals and antibodies

Antibody for RHA (Abcam Ab26271) and the blocking peptide of this antibody (Abcam Ab277786-100) were purchased from Abcam. FLI1 antibody (SC-356) and the blocking peptide (sc-356p) were purchased from Santa Cruz. IgG antibody raised in rabbit used in RIP assay was a part of MagnaRIP kit (Millipore 17-700). YK-4-279 was purchased and separated into individual enantiomers by Albany Medical Research Labs, Inc. (Albany, NY, USA).

### Recombinant protein production

Recombinant RHA protein was expressed using the Baculovirus expression system. Baculovirus stock was generated by DHX9 bacmid vector in adherent SF9 cells (Invitrogen-B82501). Rapid Titer kit (Clontech-G31406) was used to determine the multiplicity of infection (MOI) of virus stock. Virus stock at an MOI of 2.5 was used to infect 1 l of a serum-independent suspension of Sf9 cells with a cell density of 2.5 × 10^6^ cells/ml (Invitrogen-11496-015). Cells were harvested after 42–44 h of infection and all purification steps were carried out at 4°C. Whole-cell extract was prepared by homogenizing cells in ice-cold hypotonic lysis buffer (25-mM HEPES pH 7.8, 10-mM NaCl, 5-mM MgCl_2_, 0.1% Tween-20, 1-mM tris (2-carboxyethyl)phosphine [TCEP], 1-mM phenylmethylsulfonyl fluoride [PMSF] and 1 Roche protease inhibitor tablet) followed by the addition of an equal volume of hypertonic buffer (25-mM HEPES pH 7.8, 1-M NaCI, 5-mM MgCl_2_, 0.1% Tween-20, 20% Glycerol, 40-mM imidazole, 1-mM TCEP, 1-mM PMSF and 1 Roche Protease Inhibitor tablet). We incubated the lysate on ice for 30 min and then centrifuged for 10 min at 13800xg. To remove the nucleic acids, we treated the lysate with 1 mg/ml of protamine sulfate (Sigma-P4020-109) and spun the lysate at 13800xg for 15 min. Immobilized Metal Affinity Chromatography (IMAC) was performed with Nickel-charged Hi-Trap chelating columns (GE-C17-0408-01) in AKTA Explorer Chromatography (GE Healthcare). The binding buffer was composed of 25-mM HEPES pH 7.8, 250-mM NaCl, 10% glycerol, 5-mM MgCl_2_, 0.1% Tween-20, 20-mM imidazole and 1-mM TCEP. Protein fractions were eluted by continuous gradient of the binding and of the elution buffer, which was composed of 25-mM HEPES pH 7.8, 250-mM NaCl, 10% glycerol, 5-mM MgCl_2_, 0.1% Tween 20, 700-mM imidazole and 1-mM TCEP. Sodium dodecyl sulfate polyacrylamide gel electrophoresis (SDS-PAGE) was performed for the quantification of protein purity. Gels were stained with the GelCode Coomassie Blue staining kit (Pierce).

The second step of purification was carried out on a Heparin column (GE-17-0406-01) after the IMAC-eluted fraction was dialyzed to decrease the NaCl concentration to 50 mM. The binding buffer was composed of 25-mM HEPES pH7.8, 50-mM NaCl, 10% glycerol, 5-mM MgCl_2_ and 1-mM TCEP. Fractions were eluted with a continuous gradient of NaCl. SDS-PAGE, silver staining (BioRad) and Coomassie staining (Pierce) were performed to assess the protein purity.

Recombinant EWS-FLI1 was expressed in *Escherichia coli* and purified using affinity column chromatography as previously published, with the modification of the elution buffer that led to EWS-FLI1 being eluted into 200-mM NaCl and 1-M Imidazole (38).

### Helicase reaction

Infrared-labeled, double-stranded RNA (5′-IR700-GCGUCGAUCCGAAACUAUACUUAAUUUUAA-3′ and 5′-GUUUCGGAUCGACGC-3′) was synthesized and duplexed by IDTDNA. We used an unbiased random generation of an RNA sequence for this substrate given that no clear sequence preference has been reported. A Basic Local Alignment Search Tool analysis demonstrated that the UAUACUUAAUUUUAA sequence of the substrate showed homology to the CEP120 mRNA nucleotides between 4187 and 4173 and to the FAM105A mRNA nucleotides between 1704 and 1690. The calculated *Tm* value of dsRNA is 62°C. For kinetic experiments, double-stranded RNA concentrations varied from 0.125 to 32 nM. The helicase buffer was composed of 25-mM HEPES-KOH, pH 7.5, 2-mM MgCl_2_, 1-mM Dithiothreitol (DTT), 2-mM nucleoside phosphate and double-stranded RNA (39). The helicase reaction was stopped by the addition of 5× stopping buffer for a final concentration of 10% glycerol, 5-mM ethylenediaminetetraacetic acid and 0.02% Bromophenol blue.

YK-4-279 and its enantiomers were dissolved in isopropanol and pre-incubated with EWS-FLI1 for 10 min before being added to the helicase reaction. Buffer controls containing either additional protein or small molecules were included in each helicase reaction. The helicase reaction products were separated in 12% native, non-denaturant polyacrylamide gels and then visualized by an infrared scanner (Odyssey). Band intensities were determined by densitometry (ImageJ). The ratio of product to the sum of product and substrate in each lane was used to calculate the unwound product amount. All helicase data represent the average of at least three independent experiments. Graphs were generated and statistical analyses were performed by GraphPad software. Kinetic analyses were done by SigmaPlot software.

### Annealing reaction

Infrared-labeled, double-stranded RNA (5′-IR700-GCGUCGAUCCGAAACUAUACUUAAUUUUAA-3′ and 5′-GUUUCGGAUCGACGC-3′) used in the helicase reaction was heated to 95°C and then promptly transferred to ice. The annealing reaction contained 25-mM HEPES pH7.4, 1-nM-heated single-stranded RNA, 1-nM RHA, 2-mM MgCl_2_ and 1-mM DTT. The reaction was carried out at 37°C without the addition of adenosine triphosphate (ATP).

### Surface plasmon resonance

The binding affinity of EWS-FLI1 and RHA was measured using surface plasmon resonance (SPR; Biacore T-200, GE). The protein was immobilized on a CM5 carboxymethyl dextran chip with a running buffer of 10-mM HEPES (10 mM), 150-mM NaCl, 0.05% P-20. The CM5 surface was activated for 720 s by injecting an equal volume of 100-mM N-hydroxysuccinimide (NHS) and 400-mM 1-ethyl-3- (3-dimethylaminopropyl) carbodiimidehydrochloride (EDC). RHA was diluted in 10-mM sodium acetate, pH 4.0 and injected at 10 μl/min for a period of 100 s. This resulted in the attachment of RHA to the sensor surface with a density of ∼1800 response unit (RU). An injection of 1-M ethanolamine hydrochloride for 720 s deactivated any excess activated groups. The running buffer used was 10-mM HEPES, 150-mM NaCl, 0.05% P-20 (HBS-P), at a flow rate of 10 μl/min. EWS-FLI1 was used as an analyte in the running buffer in EWS-FLI1- and RHA-binding kinetic experiments. The concentrations of EWS-FLI1 in each running buffer were 24, 12, 6, 3, 1.5, 0.75 and 0.375 nM, respectively. The running buffer was composed of HBS-P containing 1-mM TCEP, 2-mM MgCl_2_ and 0.1-M imidazole, pH 7.4. The sample compartment was maintained at 10°C. SPR running conditions were as follows: flow rate, 100 μl/min; contact time, 60 s; and dissociation time, 600 s. We used 1N NaCl as the regeneration agent. The binding of EWS-FLI1 to RHA was assessed using a 1:1 Langmuir model.

### RNA-binding assay

RNA was synthesized by polymerase chain reaction and ^32^P-labeled- cytidine triphosphate (CTP) was incorporated, as previously published (40). The RNA-binding assays combined various amounts of proteins that were dissolved in buffer containing 25-mM HEPES pH 7.4, 80-mM NaCl, 2-mM MgCl_2_, 2mM-DTT and Superase In RNase inhibitor (Life Technologies AM2696) prior to adding 5 pmol ^32^P-labeled RNA. The *ets*-DNA competition assay was carried out under the above conditions in the presence of increasing concentrations of *ets*-DNA from 0 to 3125 nM (38). High-salt reactions contained 150- and 210-mM NaCl. We incubated the protein–RNA mixture for 10 min at room temperature and stopped the reaction by adding glycerol at 5% final concentration. Then, we separated free RNA from protein-bound RNA in a 6% native polyacrylamide gel in a 0.5× TBE running buffer system by electrophoresis. Gels were exposed to phosphorimager cassettes overnight and visualized via Phosphorimage scanner (Amersham). Free and protein-bound RNA in the gel images were quantified using ImageJ software (NIH).

### ATPase assay

Inorganic free phosphate was measured using the Phosphate Fluorometric assay kit (BioVision, Cat. no. K420-100). The RNA sequence used in ATPase assay was the duplex of 5′GCGUCGAUCCGAAACUAUACUUAAUUUUAA-3′ and 5′-GUUUCGGAUCGACGC-3′. The assay was carried out with 100-μM ATP according to the manufacturer's instructions.

### Atomic force microscopy

Binding reactions for RHA and EWS-FLI1 proteins were carried out with a 497-nt partially double-stranded RNA (41). The reaction mixture contained 25-mM HEPES pH 7.4, 2-mM MgCl_2_, 1-mM TCEP, dsRNA and varying concentrations of proteins. The binding reaction mixture was incubated at 25°C for 1 min. Freshly cleaved muscovite mica was incubated in a mixture of a 1-(3-aminopropyl) silatrane (APS) solution for 30 min to prepare APS-mica, as described previously (42). The sample droplets (5 μl) were deposited on APS-mica for 2 min, washed with de-ionized water and dried with nitrogen gas. The mica was attached to a metal disc with double-sided tape for imaging. Images were acquired in tapping mode in the air using a Multimode SPM Nanoscope IIIa system (Bruker/Digital Instruments, Santa Barbara, CA, USA). Silicon tapping mode probes (Hi'Res DP14; MicroMasch, Estonia) with a curvature radius apex of 1 nm were used. Nominal spring constants of ∼5.0 N/m and a resonant frequency of ∼160 Hz were used as well.

Contour-length measurements of the partially dsRNA molecule were made by tracing the image RNA backbone using the curve tool and obtaining the readout from the microscope image module (Digital Instruments, Santa Barbara, CA, USA). Protein volume measurements were performed using the section tool from the image module (Digital Instruments, Santa Barbara, CA, USA). Perpendicular cross-sections were made to record the width in two dimensions: the height (*h*) of the protein was measured by the difference in the intensity of the protein compared to the background noise; the widths were measured at half of the maximal protein height. The volume (*V*) of the protein was determined as previously described (43). The volume of the protein was converted into mass in kilodaltons, obtained from the comparison with the volumes of EWS-FLI1 and RHA (44).

The cross-sections of protein molecules to calculate the volume of protein complexes were calculated as follows:

\begin{equation*} V_c = h\pi /6(3ab/4 + h^2 ) \end{equation*}

where *V*_c_ is the molecular volume; *h* is height; and *a* and *b* are the perpendicular diameter of the protein.

### Modeling

Because there is no crystal structure for human RHA, we used homology modeling to obtain a model of this molecule. For modeling, I-Tasser (45), Swiss-Model (46) and Phyre2 (47) were used. I-Tasser and Swiss-Model were used with the default settings, while Phyre2 was used in the intensive mode. We used the entire sequence of RHA as the input for the modeling programs. All modeling packages identified structure of *S. cerevisiae* Prp43p DEAH/RHA (PDB codes: 2XAU and 3KX2) (48,49) as the template and it was used for the generation of the RHA model. The alignment of RHA and template sequences is shown in Supplementary Figure S9. COOT (50) and SSM (51) protocols were used to superpose and compare the models of RHA with the structure of superfamily 2 helicase Hel308 in complex with unwound DNA (PDB code: 2P6R) (52).

### RIP, RIP-seq and analysis

DMSO or 3-μM YK-4-279-treated TC32 cells were used for RIP analysis. We used RHA (Abcam) and FLI1 (Santa Cruz) antibodies for immunoprecipitation, which was carried out using Magna RIP (Millipore; Cat. no. 17-700) according to the manufacturer's instructions. Immunogenic peptide-blocked FLI1 and RHA antibodies were used as negative controls for RIP analysis. The extracted RNAs were sequenced on a Illumina platform by Otogenetics. The quality of RIP-seq reads from each library was assessed using FastQC (http://www.bioinformatics.babraham.ac.uk/projects/fastqc/). Reads from each RIP library were aligned to the GRCh37 genome build using Bowtie2 version 2.1.0 via TopHat version 2.0.9 (53). Fragments per kilobase of transcript per million mapped reads (FPKM) counts were generated using the recommended Cufflinks (2.2.1), Cuffmerge, and Cuffquant pipeline, followed by Cuffnorm (54). Regions with fewer than 0.5 FPKM were considered to be non-RIP, while those with greater than 0.5 FPKM were considered to be RIP. For each group (RHA and EWS-FLI1), the respective library produced by blocked antibody IP (RHA Block, FLI1 Block) was subtracted from the library produced by the unblocked antibody if FPKM at a region exceeded 0.5. We used the hypergeometric distribution test to determine the statistical significance of transcripts that overlap between RHA-RIP and EWS-FLI1-RIP (55).

## RESULTS

### Characterization of full-length recombinant RHA

**Figure 1.**
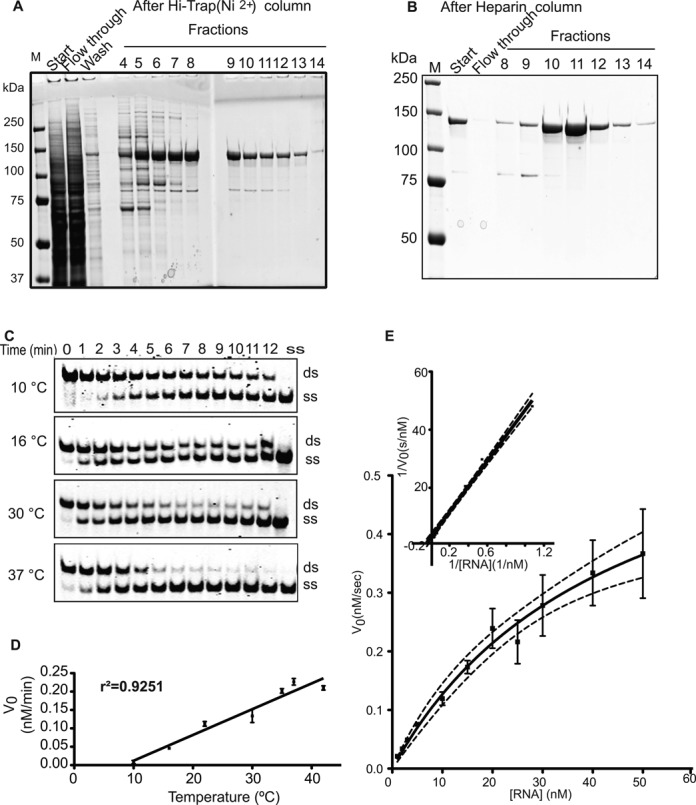
Recombinant RHA is a highly active helicase. (**A**) Eluted RHA fractions from the Hi-Trap nickel column stained with Coomassie Blue. (**B**) Heparin column-purified RHA stained with Coomassie. (**C**) One nanomolar RHA separated 1-nM dsRNA in a temperature- and time-dependent fashion to single strands. (**D**) The initial rates of the reactions were plotted against the temperatures. The linear correlation coefficient of this plot was found as *r*^2^ = 0.9251. All reactions were carried out three times. (**E**) Michaelis–Menten kinetics graph, the x-axis represents the substrate (dsRNA) concentration in nM, and the y-axis represents the initial velocity at the corresponding substrate concentration. The kinetics experiments were repeated three times and differences among individual results represented in the error bars. Inset of (E)**:** Lineweaver–Burk transformation plot of the RHA helicase enzyme reaction.

We utilized full-length RHA to avoid potential artifacts of protein interactions, such as dominant negative or enzyme substrate alterations, from the use of a truncated protein (56–58). RHA was purified from insect cells using a metal-affinity column that bound to an N-terminal His-tag followed by a heparin column in order to further reduce impurities. Heparin mimics a nucleic acid backbone (59), and in addition to the continual presence of Mg++, protein purified in this way retains its ability to properly bind nucleic acid (60). The purified RHA was 99% pure (Figure 1A and B and Supplementary Figure S1A for size exclusion chromatography results to prove the fractions used in assays contained pure RHA).

We measured the helicase activity of purified RHA by using IR700 infrared dye-labeled double-stranded RNA as a substrate in a strand separation assay (Figure 1C). RHA protein was mixed with RNA and the reaction was initiated with the addition of ATP. Control reactions with heat-inactivated RHA, as well as reactions lacking enzyme, ATP or MgCl_2_, showed no single-stranded product formation (Supplementary Figure S1B). The specific activity of RHA was calculated as 4.84 M s^−1^ ng^−1^. Our purified RHA utilizes ATP, CTP, Uridine triphosphate (UTP) and Guanosine triphosphate (GTP) in helicase reactions, as reported previously (Supplementary Figure S1C) (61). The initial velocity of RHA helicase activity is temperature dependent (Figure 1C and D) and ranges from 0.012 ± 0.003 nM/min at 10°C to 0.21 ± 0.008 nM/min at 42°C (Figure 1D) with an *r*^2^ of 0.93 for a linear temperature correlation. We obtained a value of 44-nM *K*_M_ for the helicase activity of RHA when we varied RNA concentration in the reaction (Figure 1E and F). The RHA enzyme efficiency is 1.6 × 10^8^ M^−1^ s^−1^ based on the *k*_cat_/*K*_M_, where *k*_cat_ is 0.69 s^−1^ with 1-nM initial enzyme concentration. We also assessed ATPase activity of RHA, showing that ATP hydrolysis requires the presence of RNA (Supplementary Figure S2C).

### RHA facilitates annealing of single-stranded RNA

**Figure 2.**
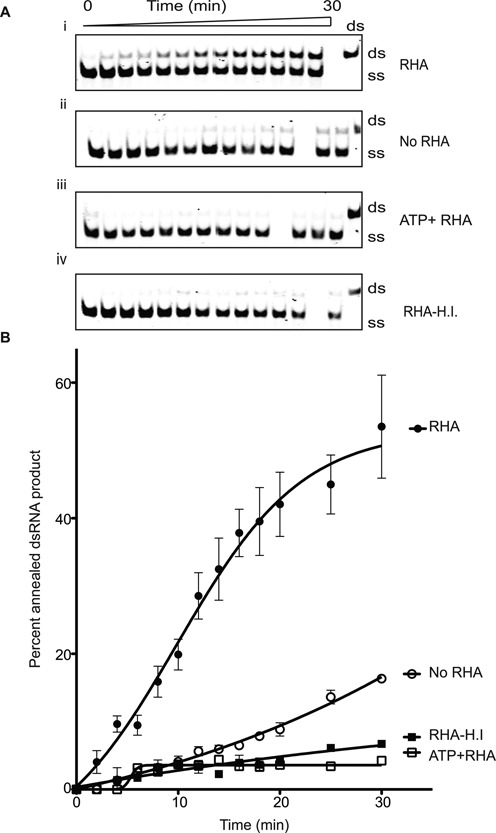
RHA facilitates the re-annealing of complementary single-stranded RNA. (**A**) i: RHA anneals single-stranded RNA to yield double-stranded products at 37°C in a time-dependent fashion. ii: annealing reaction without RHA indicated the spontaneous annealing rate of ssRNA used in the reaction. iii: RHA did not produce dsRNA in the presence of 2-mM ATP. iv: heat-inactivated RHA did not yield to dsRNA products. (**B**) The percentage of double-stranded products formed in the annealing assay was plotted against time to show the progress of product formation. All annealing assays were repeated three times.

RHA increases the annealing of tRNA (3)Lys, the primer for reverse transcription, to HIV-1 RNA (62). We therefore investigated whether our recombinant RHA possesses annealing activity using the same RNA sequences as in helicase assay but starting as a single-strand with a 1:1 molarity ratio to RHA. In the absence of ATP, RHA facilitated the annealing RNA to yield dsRNA with an efficiency of 50% at 37°C; this reaction was inhibited by 2-mM ATP (Figure 2A). Spontaneous annealing of RNA without RHA resulted in only 15% dsRNA (Figure 2B) while heat-inactivating RHA did not yield any dsRNA product (Figure 2B).

### EWS-FLI1 reduces RHA helicase activity

**Figure 3.**
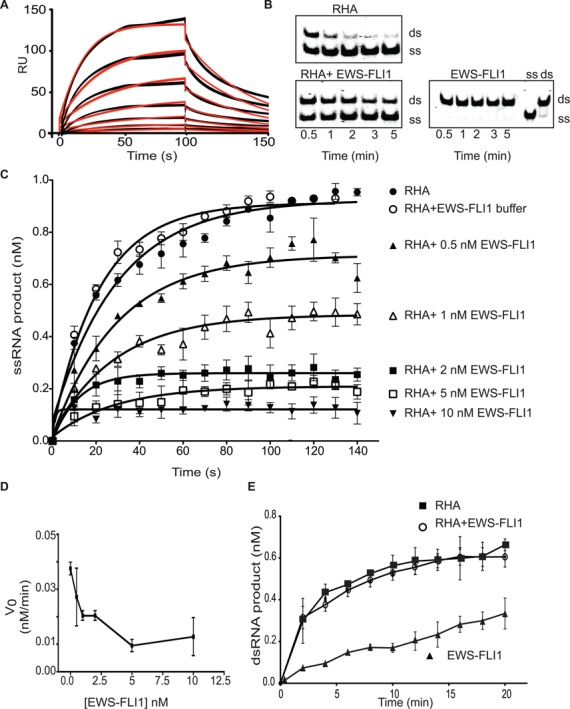
EWS-FLI1 interferes with the helicase activity of RHA. (**A**) EWS-FLI1 interacts directly with full-length RHA using surface plasmon resonance. Sensorgrams show a range of EWS-FLI1 concentrations (from 24 to 0.374 nM) in black lines. The model fits of these triplet values are represented by the red curves. The x-axis is the interaction time in seconds. The y-axis is the relative response unit (RU). The graph represents one of the nine independent experiments. (**B**) EWS-FLI1 inhibits the helicase activity of RHA. The upper band shows dsRNA and the lower band shows ssRNA. One nanomolar RHA shows complete unwinding (the upper gel) and 1-nM RHA was combined with 1-nM EWS-FLI1 that inhibited unwinding (lower gel). 1-nM EWS-FLI1 alone does not catalyze the helicase reaction (lower right gel). dsRNA and ssRNA were loaded to the gel as size markers (on the right). (**C**) Increasing concentrations of EWS-FLI1 were mixed with RHA in the helicase reaction. Single-stranded product formation of the reactions over time was plotted using the key: open circles (○) buffer control; closed circles (•) RHA alone; black triangle (▴) 0.5-nM EWS-FLI1; open triangle (Δ) 1-nM EWS-FLI1; black square (▪) 2-nM EWS-FLI1; open square (□) 5-nM EWS-FLI1; and black downward triangle (▾) 10-nM EWS-FLI1. (**D**) EWS-FLI1 affected the initial velocity of the RHA helicase reaction in a dose-dependent fashion. The x-axis shows the EWS-FLI1 concentration (nM), and the y-axis shows the initial velocity of the helicase reaction. (**E**) EWS-FLI1 did not interfere with the annealing function of RHA. The annealing reaction was carried out with 1-nM RHA—black square (▪)—or 1-nM RHA with 1-nM EWS-FLI1—open circle (○)—or 1-nM EWS-FLI1—black triangle (▴). The reaction was 20 min and the time course is plotted as dsRNA product (nM) versus time (min). Annealing experiments were carried out three times.

Having established the biochemical properties of our recombinant RHA, we evaluated the effect of adding EWS-FLI1 to the helicase assay. We previously reported that an RHA_647–1075aa_ fragment and EWS-FLI1 bound directly (33,34), but at that time did not have pure full-length RHA for binding nor kinetic measurements. We immobilized full-length RHA on a CM5 chip and used purified EWS-FLI1 (38) (Supplementary Figure S2A) as an analyte to quantify the interaction between the two proteins using SPR. The dissociation constant *K*_d_ was 0.030 ± 0.01 s^−1^ and the association constant (*k*_a_) was 3.5 × 10^6^ ± 1.9 × 10^6^ M^−1^ s^−1^ leading to a calculated affinity of 14 ± 4.6 nM for the two proteins (Figure 3A). These binding experiments showed that full-length RHA and EWS-FLI1 interact rapidly and with high affinity.

In order to test the effect of EWS-FLI1 on RHA helicase activity, we used varying molar ratios of both proteins in the helicase reaction that was initiated with the addition of ATP. EWS-FLI1 reduced the strand separation of dsRNA catalyzed by RHA in a dose-dependent fashion (Figure 3B and C). When EWS-FLI1 and RHA are present in a 1:1 stoichiometric concentration of 1 nM, we measured a 58% reduction in initial helicase velocity. By increasing EWS-FLI1 to 10 nM, the initial velocity was reduced by 78% of control, demonstrating that the initial velocity of RHA is inversely correlated with the concentration of EWS-FLI1 in the reaction (Figure 3D). EWS-FLI1 inhibition of RHA helicase activity does not follow standard Michaelis–Menten kinetics, suggesting a complex model.

Although 1-nM EWS-FLI1 caused a 50% reduction in the helicase activity of RHA, it did not hinder the time-dependent annealing function of RHA (Figure 3E). In fact, the increased concentration of EWS-FLI1 facilitated RHA annealing of RNA using a constant RHA concentration during a 2-min incubation (Supplementary Figure S2B). We observed that 1-nM EWS-FLI1, alone, annealed RNA in a time-dependent fashion (Figure 3E). In contrast to the effect upon helicase activity, EWS-FLI1 does not affect the ATPase activity of RHA (Supplementary Figure S2C).

### EWS-FLI1 binds to RNA

**Figure 4.**
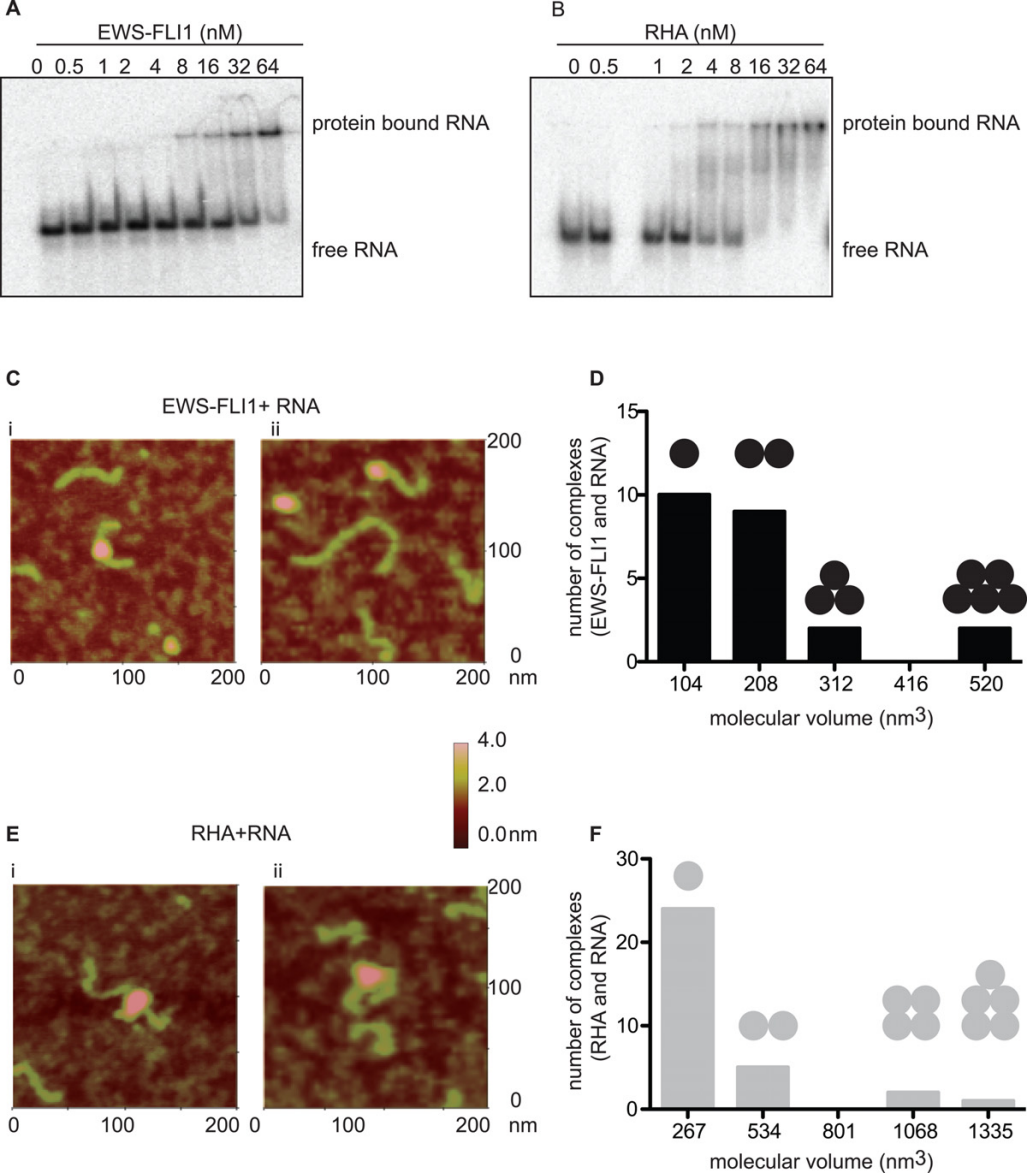
EWS-FLI1 binds to RNA. (**A**) Concentrations from 0.5 to 64 nM of EWS-FLI1 bind to ^32^P-labeled 350-nt long dsRNA in gel shift assay, representative of three experiments. (**B**) Concentrations from 0.5 to 64 nM of RHA bind to ^32^P-labeled 350-nt long dsRNA in gel shift assay, representative of three experiments. (**C**) EWS-FLI1 binds to dsRNA. i–ii: AFM images of RNA-bound EWS-FLI1. The green object is dsRNA and the pink object is EWS-FLI1 protein. The color scale given on the right side of the image represents the height of the protein on the surface. (**D**) The volume distribution of RNA bound EWS-FLI1 (as the calculated volume of EWS-FLI1 was 104 nm^3^) protein complexes. The number of black spheres on the bar graphs represents the number of EWS-FLI1 protein on RNA complexes. (**E**) RHA binds to RNA. i–ii: AFM images of RNA-bound RHA. The green object is dsRNA and the pink object is RHA protein. (**F**) The volume distribution of RNA-bound RHA (as the calculated volume of EWS-FLI1 was 267 nm^3^) protein complexes. The number of gray spheres on the bar graphs represents the number of RHA proteins on RNA complexes.

In order to further understand the inhibitory mechanism of EWS-FLI1 on helicase activity and the facilitating effect on the annealing activity of RHA, we tested whether EWS-FLI1 binds to RNA. We used a 350-nt partially dsRNA from hepatitis delta virus and combined this with either RHA or EWS-FLI1 followed by native PAGE resolution of complexes. This RNA was selected as a ‘non-specific’ potential ligand for EWS-FLI1. EWS-FLI1 binding to RNA was confirmed with a *k*_d apparent_ of 30 ± 3 nM (Figure 4A). RHA bound RNA with a dissociation constant of *k*_d apparent_ 4.7 ± 0.2 nM (Figure 4B). To support our conclusion that this binding is not an artifact of buffer conditions, we increased the salt concentration of the RNA binding buffer to 210-nM NaCl and measured only a 3-fold decrease in affinity (Supplementary Figure S3).

In order to further demonstrate our novel finding that EWS-FLI1 directly binds to RNA, we utilized AFM imaging. We used a 497-nt partially dsRNA sequence from hepatitis delta virus (497L RNA) (41), 65 nm in length by AFM. This RNA was selected since it was of a defined length for AFM measurements and long enough for multiple protein–RNA interactions to occur but we were agnostic to the sequence. The theoretical individual volumes of EWS-FLI1 and RHA were calculated as 104 nm^3^ and 267 nm^3^, respectively. We show two examples of RNA directly bound to EWS-FLI1 using AFM (Figure 4C(i–ii)). Using the volumetric measurement of EWS-FLI1, we identified 44% of the complexes of EWS-FLI1 bound to RNA in 1:1 and 39% of those bound to RNA in 2:1 stoichiometric ratios (Figure 4D). RHA binding to RNA was also measured by AFM, however, in contrast to EWS-FLI1, 75% were bound as monomeric complexes (Figure 4E(i–ii) and F).

We modeled the RNA-binding ability of EWS-FLI1 using RNA-binding prediction applications such as BindN (63), RNABindR (64) and catRAPID (65). These algorithms corroborated a minimally common predicted region of the *ets-*DNA-binding domain RNA binding (Supplementary Figure S4A–C, the boxed region indicates the *ets*-DNA binding domain). To confirm these predictive models, a gel-shift analysis showed that the addition of a wild-type *ets* DNA oligodeoxynucleotide (ODN) competed with the RNA-binding ability of EWS-FLI1; however, a mutant *ets* ODN was only 30% as efficient as the wild-type at displacing the RNA (Supplementary Figure S5).

### YK-4-279 reverses EWS-FLI1 inhibition of RHA helicase activity

**Figure 5.**
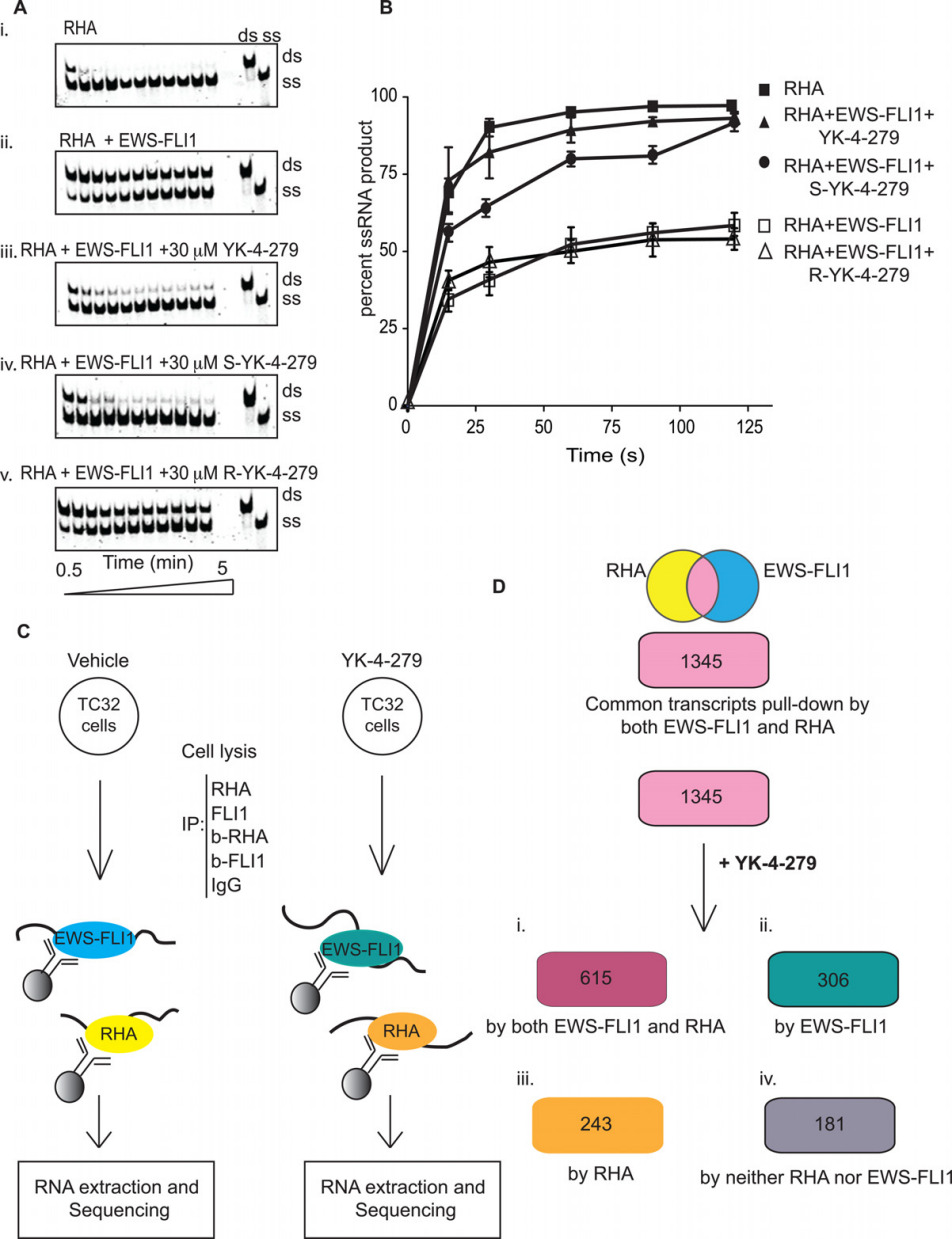
YK-4-279 negates the inhibitory effect of EWS-FLI1 on RHA in an enantiomer-specific manner and alters the profile of bound RNA. (**A**) Recombinant RHA (1 nM) in the presence of recombinant EWS-FLI1 (1 nM) or small molecules (30 μM). Helicase reactions carried out as shown above in Figure 1. (**B**) Triplicate results of helicase reactions were plotted over time. The x-axis is time in seconds and the y-axis is percent product formation in the helicase assay. (**C**) Scheme used for RNA immunoprecipitation (RIP) of RHA or EWS-FLI1 protein complexes. TC32 ES cells either treated with DMSO or 3-μM YK-4-279 for 8 h. Extraction of RNA associated with RHA or EWS-FLI1 complexes was followed by Illumina sequencing. (**D**) 1345 transcripts were found common in RHA or EWS-FLI1 RIP. YK-4-279 changes the profile of 1345 RNA bound to the proteins. i: after YK-4-279 treatment, 615 of 1345 RNAs were immunoprecipitated separately by either RHA or EWS-FLI1 antibodies. ii: 306 transcripts were immunoprecipitated by EWS-FLI1. iii: RHA RIP yielded 243 transcripts. iv: 181 transcripts were not found in sequencing from either IP reaction.

Our previous publications showed that YK-4-279 inhibited RHA immunoprecipitation with EWS-FLI1 from ES cell lysates in an enantiomer-specific fashion (35). We therefore evaluated the effect of YK-4-279 on the EWS-FLI1-dependent inhibition of RHA helicase activity. Both racemic YK-4-279 and (S)-YK-4-279 disinhibited the helicase reaction, showing restoration of 80% helicase activity in the presence of 1-nM EWS-FLI1 (Figure 5A(iii–iv) and B). Consistent with prior enantiospecific immunoprecipitation results, (R)-YK-4-279 did not restore helicase activity (Figure 5A(v) and B) (35,66). YK-4-279 alone did not alter RHA helicase activity (Supplementary Figure S6). Since YK-4-279 reverses the inhibitory effect of EWS-FLI1 upon RHA activity investigated whether YK-4-279 alters RNA binding to either protein.

### EWS-FLI1 and RHA immunoprecipitated RNA is altered by YK-4-279

We queried the profiles of RNA binding to EWS-FLI1 and RHA complexes in ES and whether this would change following treatment with YK-4-279. We performed RIP using ES TC32 cells treated with either vehicle (DMSO) or 3-μM YK-4-279 for 8 h followed by cell lysis and immunoprecipitation of protein complexes (Figure 5C). EWS-FLI1 or RHA antibodies precipitated RNA associated with either protein followed by RNA extraction. Extracted RNA was transcribed to cDNA and sequenced using an Illumina platform. DNA sequences were aligned to the GRCh37 genome and transcript FPKM was calculated. Transcripts that immunoprecipitated in samples treated with blocking peptides for either antibody were removed from analysis as nonspecific. We also excluded the transcripts having lower than 0.5 FPKM values from the analysis in order to increase stringency.

Alignment of sequencing results showed that 6.9% of the total cellular RNA was uniquely present in the RHA immunoprecipitation, while 4.8% was unique to the EWS-FLI1 RIP. The remaining 86.9% of unique cellular RNA sequences were not immunoprecipitated in either condition. We determined RIP from either EWS-FLI1 or RHA contained 1345 overlapping transcripts (Figure 5C, Supplementary Table 1). These overlapping transcripts were statistically significant (*P* = 2.208 x 10^−296^) by using the hypergeometric distribution test. Gene Ontology term enrichment (GO) analysis by GOrilla (67,68) classified RNA found in both RHA and EWS–FLI1 protein complexes as enriched for transcripts categorized in the following: cell–cell adhesion, inflammatory response, negative regulation of vasculature development, regulation of vasoconstriction, sensory perception, neuronal action potential, humoral immune response, cell–cell signaling and negative regulation of Notch signaling (Supplementary Figure S7A). The results of GO term analysis suggest that there is a significant probability that cellular functional pathways are perturbed by changes in RNA metabolism when EWS-FLI1 binds to RHA.

This GO term analysis led us to evaluate YK-4-279-treated cells for RNA binding profile changes. We analyzed our data based on four subsets of isolated RNA (i) those that continued to be overlapping from both RIP (46% of the transcripts), (ii) those only present with EWS-FLI1 RIP (23% of the transcripts), (iii) those only present with RHA RIP (18% of the transcripts) and (iv) those RNA which were no longer RIP by either protein (13% of the transcripts) (Figure 5D). RNA bound only to EWS-FLI1 following YK-4-279 treatment was enriched for single organism signaling and cell communication (*P* = 5.7 × 10^−4^ and 4.7 × 10^−4^, respectively; Supplementary Figure S7C). The GO term enrichment analysis of RNAs bound only to RHA was enriched for the negative regulation of megakaryocyte differentiation and Histone H4K20 demethylation (*P* = 3.3 × 10^−4^ and 6.0 × 10^−4^, respectively; Supplementary Figure S7D). Thirteen percent of the RNA present in the overlapping RIP profile were no longer present in EWS-FLI1 nor RHA RIP. These RNAs that were ‘lost’ from the protein complexes following YK-4-279 treatment were enriched for JAK-STAT signaling cascade (*P* = 8.4 × 10 ^-4^; Supplementary Figure S7E). These data show that a common collection of RNA RIP independently by either EWS-FLI1 or RHA exists. Furthermore, YK-4-279 treatment leads to shifts in the RIP profiles that affect many cellular pathways.

We directly evaluated YK-4-279 for its ability to directly dissociate either EWS-FLI1 or RHA from a complex with RNA. Neither EWS-FLI1 nor RHA binding to a 350-nt-long-radiolabeled dsRNA containing 3 GGAA repeats was altered following YK-4-279 treatment in solution (Supplementary Figure S8A). This result was confirmed by titrating the amount of recombinant EWS-FLI1 or RHA in the reaction (Supplementary Figure S8B).

### *In silico* modeling the EWS-FLI1 effect upon RHA activity

Our published studies demonstrated that EWS-FLI1 binds to RHA adjacent to its helicase domain (33). There is no published crystal structure of full-length RHA containing the region of RHA amino acids between 647 and 1075 where RHA binds to EWS-FLI1. Thus, we created an *in silico* model based upon homologous sequences to *S. cerevisiae* Prp43p DEAH/RHA (PDB codes: 2XAU and 3KX2) (48,49). Prp43p has 26% sequence identity to a human DEAD box family member DDX15, which is closely related to RHA. Therefore, a model of the fragment containing residues 300–1100 may therefore be reliably constructed. Fortunately, the RHA residues previously identified as binding to EWS-FLI1, aa 822–831, were present in the model based upon the Prp43p structure.

**Figure 6.**
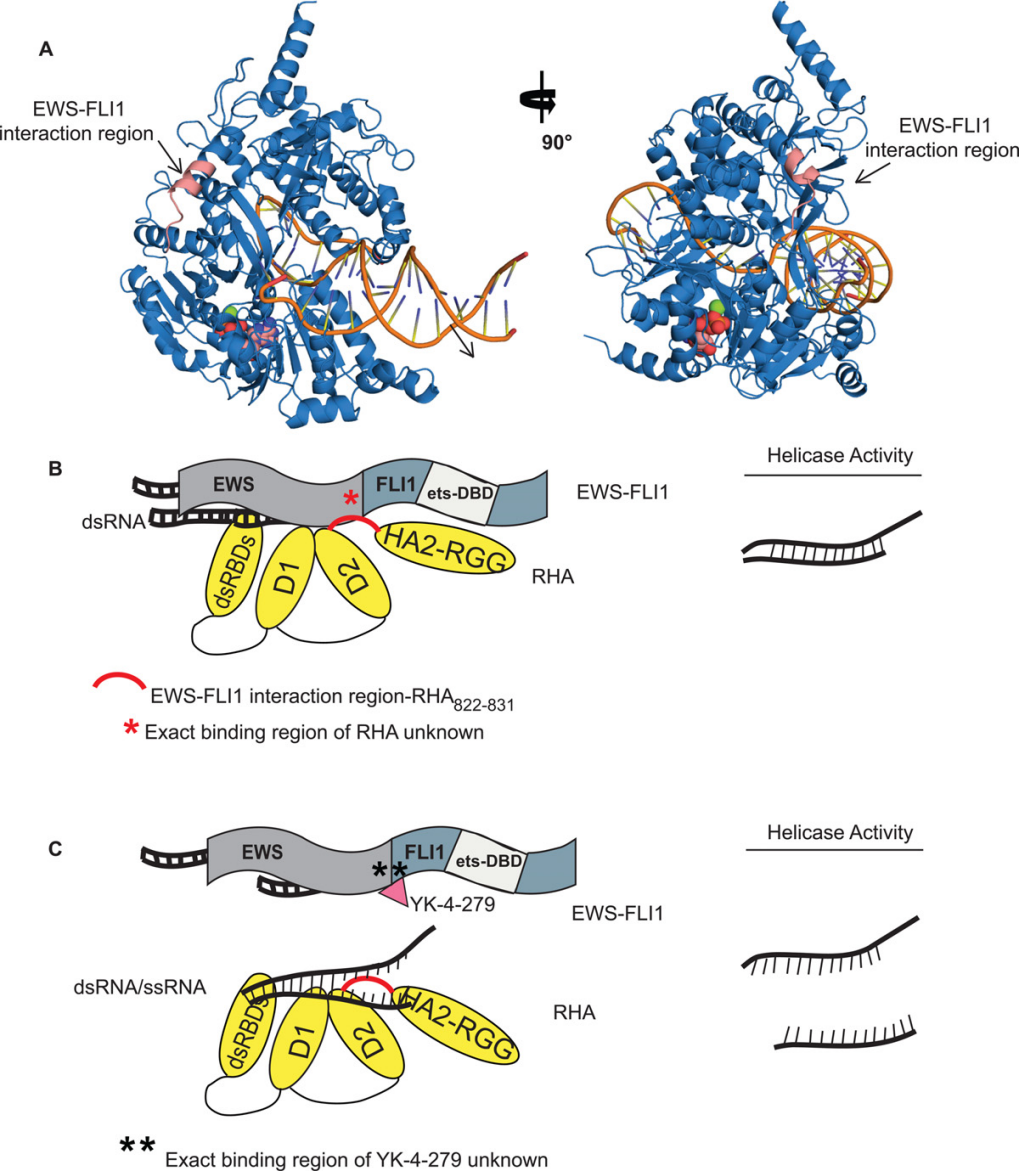
Modeling the EWS-FLI1 effect upon RHA activity. (**A**) Structure of RHA calculated using homology modeling and structure of *S. cerevisiae* Prp43p as a template. DNA position was modeled based on superimposition of the RHA model with the structure of superfamily 2 helicase Hel308 in complex with unwound DNA (PDB code: 2P6R). The model with 90° right turn is shown in the successive image. The orange indicates unwound DNA. The salmon colored residues demonstrate the EWS-FLI1 binding region of RHA. The green, and pink and red space filling molecules indicate MgCl_2_ and ADP, respectively. (**B**) The cartoons of RHA and EWS-FLI1 interaction explain the working hypothesis. RHA consists of dsRBDs, Helicase Domain 1 (D1), Helicase Domain 2 (D2) and HA2 with RGG domains. EWS-FLI1 consists of N-terminal domain of EWS and C-terminal domain of FLI1 (containing the ets-DNA binding domain, ets-DBD). EWS-FLI1 interacts with RHA amino acids between 822 and 832 shown in red. Helicase activity is reduced when EWS-FLI1 binds to RHA. However, the exact binding region of RHA upon EWS-FLI1 is unknown. (**C**) Following the addition of YK-4-279 shown in the red triangle, the helicase activity of RHA resumes to wild-type activity. Of note, the exact binding region of YK-4-279 is unknown.

A homology model using the protein family (Pfam) database (69) classified motifs DEAD/DEAH box helicase (residues 393–546; PF00270), helicase conserved C-terminal domain (residues 679–767; PF00271) and helicase-associated domain—HA2 (residues 830–918; PF04408) (Supplementary Figure S9). The conserved helicase motifs of our modeling superimposed with those of the template of Prp43p (Supplementary Figure S10). The model indicates that EWS-FLI1 binds to RHA (salmon ribbon structure) on the opposite side of the protein as nucleic acids, while green and red represent ionic interactions (Figure 6A). The quality scores for the best-obtained models were as follows: I-Tasser (TM-score: 0.50 ± 0.15), Swiss-Model (QMEAN Z-score: −4.45) and Phyre2 (confidence in the model: 964 residues (76%) modeled at >90% accuracy). The authors of I-Tasser show that a TM-score >0.5 indicates a model of correct topology and a TM-score <0.17 indicates random similarity. Thus, the indicators from both I-Tasser and Phyre2 support the likelihood that our predicted models have the correct overall folding for the core RHA fragment, and provide a robust model for a structural explanation of our experimental results. This model shows that the EWS–FLI1 interaction site is located on the reverse side of the nucleic acid interaction region. According to our model, the residues in the predicted RHA-EWS-FLI1 binding site are solvent-exposed and accessible for EWS–FLI1 interaction.

We observed that EWS-FLI1 inhibited only RHA unwinding activity and this is reversed by YK-4-279. Our working hypothesis suggests that when EWS-FLI1 bound to RHA, RHA enzymatic activity is sterically hindered leading to a lower efficiency reaction (Figure 6B). However, when YK-4-279 disrupts the complex in cells, RHA helicase activity can proceed with less impedance (Figure 6C).

## DISCUSSION

The role of RHA in diverse cellular processes is modulated by its intrinsic enzymatic activity and/or its role as a protein scaffold. Our goal was to determine the effects of EWS-FLI1 upon RHA helicase activity since prior data identified EWS-FLI1 binds to RHA near its helicase domain. We produced full-length, recombinant RHA and characterized it as having a high specific activity for unwinding RNA as well as reannealing properties. We demonstrated that EWS-FLI1 inhibited the helicase activity of RHA *in vitro* and that YK-4-279, which blocks the interaction between EWS-FLI1 and RHA, reversed the inhibitory effect. In this study, we also report a novel, unexpected finding that EWS-FLI1 can bind to RNA with physiological affinity. Finally, we show a possible mechanism connecting the altered RHA activity with ES biology based upon a significant shift in the RNA binding profiles of both EWS-FLI1 and RHA complexes when disrupted by YK-4-279.

Our full-length recombinant RHA exhibited high RNA helicase activity permitting the usage of dsRNA substrate to probe the functional effect of EWS-FLI1 on RHA activity. Our analysis of RHA kinetics, in the presence of EWS-FLI1, suggested the existence of an inhibitory mechanism that does not fit standard models. We showed that EWS-FLI1 directly binds to RNA as a possible means to block helicase enzyme activity without affecting ATPase activity. This conclusion is supported by a recent publication showing that RHA has a unique unwinding mechanism linking unwinding with reannealing cycles (70). A single RHA molecule can repeat unwinding, stalling and reannealing steps without dissociation from an RNA molecule. We show that EWS-FLI1 inhibition on unwinding activity did not reduce the reannealing rate of single-stranded substrates of RHA. In fact, EWS-FLI1 facilitated annealing of single-stranded RNA to form dsRNA in a dose-dependent manner. The unwound RNA in the presence EWS-FLI1 may impede RHA function. An alternative conclusion is that the inhibitory effect of EWS-FLI1 may simply be attributable to competition with RHA that neutralizes the electrostatic interaction of RHA with nucleic acids. Resolution of these questions requires investigating the effects of EWS-FLI1 on the modulation of repetitive sub-steps of RHA activity.

EWS-FLI1 binds to RHA amino acids between 822 and 831 in a region that is just distal to the Helicase Associated 2 (HA2) domain, which is an essential domain for helicase activity (71). As predicted by *in silico* modeling, the conformational changes posed due to EWS-FLI1 binding to this region might reduce the helicase activity of RHA. The deletion of the HA2 domain did not affect the ATPase activity of RHA (71), which is also consistent with our finding that EWS-FLI1 altered unwinding activity without any effect on ATPase activity of RHA. The ATPase activity of MLE is sufficient for the transcription of targeted genes, whereas the helicase activity of MLE is necessary for distributing the MSL complex along the X-chromosome in *Drosophila* (72). Therefore, the functional ATPase and helicase activities of RHA are essential for the physiological role of MLE (73). Overall, the literature along with our investigations, supports a dissociation of RHA ATPase and helicase activity that would lead to a different functional outcome. In the case of EWS-FLI1, this disconnect will be explored in future work as a novel mechanism of oncogenesis.

Gel-shift assays and AFM corroborated in leading us to a novel conclusion that EWS-FLI1 binds to RNA. The RNA binding of EWS-FLI1 could be monomeric or dimeric as seen in AFM results. This simple stoichiometric binding could be the result of the RNA species used in these assays. These results challenge a current paradigm of EWS-FLI1 as strictly a DNA-binding protein; since the RNA-binding domain of EWS is lost in the translocation and FLI1 has never been described as binding to RNA (74). Examples of DNA binding domains also binding RNA are found in the Drosophila homeodomain protein bicoid that binds both DNA and RNA through a helix-turn-helix structure (75). In addition to its function as a transcription factor, the bicoid protein has control over the translation of certain RNA products due to its RNA binding feature (75,76). The structural similarity between the DNA-binding domain of bicoid and the *ets* proteins support our findings (77). It is possible that regions of EWS-FLI1 outside of the DNA binding domain also bind to RNA; future work will investigate and resolve these possibilities. The RNA binding of EWS-FLI1 also suggests future investigations to evaluate whether the oncogenic effect of EWS-FLI1 could be through RNA metabolism, in addition to its DNA binding ability and transcript activation properties.

The use of a small molecule to probe the effects of the protein interaction of EWS-FLI1 with RHA provided insights into potential mechanisms of inhibition. While YK-4-279 restored the helicase activity of RHA, YK-4-279 did not change the affinity of individual proteins to RNA. After YK-4-279 treatment of ES cells, approximately half of the common transcripts found in either the EWS-FLI1 or RHA protein complexes were retained as common RNAs. Therefore, the protein–protein interactions that are disrupted by YK-4-279 appear to affect both the profile of RNA binding and the processing of the retained RNA. A recent publication showed a direct effect of the splicing protein Prp8 upon the helicase activity of Brr2 where the mechanism is due to competition for RNA substrate (78). In an additional example, Upf1, a key helicase in nonsense-mediated decay, shows decreased affinity for RNA when Upf2 binds, leading to an increase in helicase activity (79). Thus, other biologic systems support our conclusions favoring an inhibitory mechanism of EWS-FLI1 on RHA helicase activity as complex and at least in part due to both the protein–protein interaction and RNA interactions with the two proteins.

The RIP assay confirmed the presence of RNA in the EWS–FLI1 protein complexes, which might or might not contain RHA. It is possible that EWS-FLI1 and RHA bind to the same RNA either simultaneously or sequentially. Due to the technical inability to tandem IP for the RIP assay, all the overlapping RNAs found in EWS-FLI1 or RHA immunoprecipitation carry the equal possibility of being shared or bound separately by respective proteins. We did find that YK-4-279, by blocking RHA from binding to EWS-FLI1, changed the composition of the overlapping RNA molecules identified. Further, the GO analysis demonstrated a shift in the functional profiles of RNA when EWS-FLI1 binds to RHA or when the complex is disrupted with EWS-FLI1. This experiment suggests that the role of helicases in oncogenesis requires additional investigation to evaluate whether helicases are critical cancer proteins.

This publication is the first to show that targeting an oncogenic protein–protein interaction by a small molecule restores wild-type RNA helicase activity without changing RNA-binding affinity of the respective proteins. It is important to recognize that our current results show that EWS-FLI1 significantly reduces, but does not fully eliminate helicase activity. Thus, reduced helicase activity may alter RNA metabolism with subsequent effects on the overall proteome leading to oncogenesis. This is consistent with our prior studies showing that an ATP-binding mutant RHA, devoid of helicase activity, caused decreased oncogenic growth of ES cells while (S)-YK-4-279 led to apoptotic cell death (33,35,66).

Our current data show that EWS-FLI1 inhibits helicase activity of RHA and this inhibition is reversed only by (S)-YK-4-279; this suggests a novel role for EWS-FLI1 in RNA processing. We can only hypothesize based upon these data that EWS-FLI1 and RHA regulation of RNA transcripts are part of a complex oncogenic mechanism. The contribution of this interaction toward oncogenesis may be independent or related to the overlapping profile of EWS-FLI1 and RHA RIP transcripts. Further studies are needed that dissect the potentially oncogenic role of EWS-FLI1 upon RHA helicase activity based upon modulation of specific RNA or pathways. In addition, clarifying the critical balance between the scaffolding function of a helicase and the modulation of its helicase activity will shed light on the process of oncogenesis while potentially identifying new therapeutic targets.

## SUPPLEMENTARY DATA

Supplementary Data are available at NAR Online.

SUPPLEMENTARY DATA

## References

[1] Zhang Z., Pugh B.F. (2011). High-resolution genome-wide mapping of the primary structure of chromatin. Cell.

[2] Sumazin P., Yang X., Chiu H.S., Chung W.J., Iyer A., Llobet-Navas D., Rajbhandari P., Bansal M., Guarnieri P., Silva J. (2011). An extensive microRNA-mediated network of RNA-RNA interactions regulates established oncogenic pathways in glioblastoma. Cell.

[3] Cordin O., Hahn D., Beggs J.D. (2012). Structure, function and regulation of spliceosomal RNA helicases. Curr. Opin. Cell Biol..

[4] Bohnsack M.T., Martin R., Granneman S., Ruprecht M., Schleiff E., Tollervey D. (2009). Prp43 bound at different sites on the pre-rRNA performs distinct functions in ribosome synthesis. Mol. Cell.

[5] Pisareva V.P., Pisarev A.V., Komar A.A., Hellen C.U., Pestova T.V. (2008). Translation initiation on mammalian mRNAs with structured 5′UTRs requires DExH-box protein DHX29. Cell.

[6] Chen C.Y., Liu X., Boris-Lawrie K., Sharma A., Jeang K.T. (2013). Cellular RNA helicases and HIV-1: insights from genome-wide, proteomic, and molecular studies. Virus Res..

[7] Guil S., Esteller M. (2012). Cis-acting noncoding RNAs: friends and foes. Nat. Struct. Mol. Biol..

[8] Bleichert F., Baserga S.J. (2007). The long unwinding road of RNA helicases. Mol. Cell.

[9] Botlagunta M., Vesuna F., Mironchik Y., Raman A., Lisok A., Winnard P., Mukadam S., Van Diest P., Chen J.H., Farabaugh P. (2008). Oncogenic role of DDX3 in breast cancer biogenesis. Oncogene.

[10] Chang P.C., Chi C.W., Chau G.Y., Li F.Y., Tsai Y.H., Wu J.C., Wu Lee Y.H. (2006). DDX3, a DEAD box RNA helicase, is deregulated in hepatitis virus-associated hepatocellular carcinoma and is involved in cell growth control. Oncogene.

[11] Tanaka N., Aronova A., Schwer B. (2007). Ntr1 activates the Prp43 helicase to trigger release of lariat-intron from the spliceosome. Genes Dev..

[12] Hilbert M., Kebbel F., Gubaev A., Klostermeier D. (2011). eIF4G stimulates the activity of the DEAD box protein eIF4A by a conformational guidance mechanism. Nucleic Acids Res..

[13] Schutz P., Bumann M., Oberholzer A.E., Bieniossek C., Trachsel H., Altmann M., Baumann U. (2008). Crystal structure of the yeast eIF4A-eIF4G complex: an RNA-helicase controlled by protein-protein interactions. Proc. Natl. Acad. Sci. U.S.A..

[14] Lebaron S., Papin C., Capeyrou R., Chen Y.L., Froment C., Monsarrat B., Caizergues-Ferrer M., Grigoriev M., Henry Y. (2009). The ATPase and helicase activities of Prp43p are stimulated by the G-patch protein Pfa1p during yeast ribosome biogenesis. EMBO J..

[15] Young C.L., Khoshnevis S., Karbstein K. (2013). Cofactor-dependent specificity of a DEAD-box protein. Proc. Natl. Acad. Sci. U.S.A..

[16] Dossani Z.Y., Weirich C.S., Erzberger J.P., Berger J.M., Weis K. (2009). Structure of the C-terminus of the mRNA export factor Dbp5 reveals the interaction surface for the ATPase activator Gle1. Proc. Natl. Acad. Sci. U.S.A..

[17] Bolger T.A., Folkmann A.W., Tran E.J., Wente S.R. (2008). The mRNA export factor Gle1 and inositol hexakisphosphate regulate distinct stages of translation. Cell.

[18] Miller A.L., Suntharalingam M., Johnson S.L., Audhya A., Emr S.D., Wente S.R. (2004). Cytoplasmic inositol hexakisphosphate production is sufficient for mediating the Gle1-mRNA export pathway. J. Biol. Chem..

[19] von Moeller H., Basquin C., Conti E. (2009). The mRNA export protein DBP5 binds RNA and the cytoplasmic nucleoporin NUP214 in a mutually exclusive manner. Nat. Struct. Mol. Biol..

[20] Lee C.G., Hurwitz J. (1992). A new RNA helicase isolated from HeLa cells that catalytically translocates in the 3′ to 5′ direction. J. Biol. Chem..

[21] Lee C.G., da Costa Soares V., Newberger C., Manova K., Lacy E., Hurwitz J. (1998). RNA helicase A is essential for normal gastrulation. Proc. Natl. Acad. Sci. U.S.A..

[22] Lee C.G., Hurwitz J. (1993). Human RNA helicase A is homologous to the maleless protein of Drosophila. J. Biol. Chem..

[23] Kuroda M.I., Kernan M.J., Kreber R., Ganetzky B., Baker B.S. (1991). The maleless protein associates with the X chromosome to regulate dosage compensation in Drosophila. Cell.

[24] Anderson S.F., Schlegel B.P., Nakajima T., Wolpin E.S., Parvin J.D. (1998). BRCA1 protein is linked to the RNA polymerase II holoenzyme complex via RNA helicase A. Nat. Genet..

[25] Aratani S., Fujii R., Oishi T., Fujita H., Amano T., Ohshima T., Hagiwara M., Fukamizu A., Nakajima T. (2001). Dual roles of RNA helicase A in CREB-dependent transcription. Mol. Cell. Biol..

[26] Nakajima T., Uchida C., Anderson S.F., Lee C.G., Hurwitz J., Parvin J.D., Montminy M. (1997). RNA helicase A mediates association of CBP with RNA polymerase II. Cell.

[27] Hartman T.R., Qian S., Bolinger C., Fernandez S., Schoenberg D.R., Boris-Lawrie K. (2006). RNA helicase A is necessary for translation of selected messenger RNAs. Nat. Struct. Mol. Biol..

[28] Mills J.R., Malina A., Lee T., Di Paola D., Larsson O., Miething C., Grosse F., Tang H., Zannis-Hadjopoulos M., Lowe S.W. (2013). RNAi screening uncovers Dhx9 as a modifier of ABT-737 resistance in an Emu-myc/Bcl-2 mouse model. Blood.

[29] Jain A., Bacolla A., Del Mundo I.M., Zhao J., Wang G., Vasquez K.M. (2013). DHX9 helicase is involved in preventing genomic instability induced by alternatively structured DNA in human cells. Nucleic Acids Res..

[30] Jain A., Bacolla A., Chakraborty P., Grosse F., Vasquez K.M. (2010). Human DHX9 helicase unwinds triple-helical DNA structures. Biochemistry.

[31] Chakraborty P., Grosse F. (2010). WRN helicase unwinds Okazaki fragment-like hybrids in a reaction stimulated by the human DHX9 helicase. Nucleic Acids Res..

[32] Delattre O., Zucman J., Melot T., Garau X.S., Zucker J.M., Lenoir G.M., Ambros P.F., Sheer D., Turc-Carel C., Triche T.J. (1994). The Ewing family of tumors—a subgroup of small-round-cell tumors defined by specific chimeric transcripts. N. Engl. J. Med..

[33] Toretsky J.A., Erkizan V., Levenson A., Abaan O.D., Parvin J.D., Cripe T.P., Rice A.M., Lee S.B., Uren A. (2006). Oncoprotein EWS-FLI1 activity is enhanced by RNA helicase A. Cancer Res..

[34] Erkizan H.V., Kong Y., Merchant M., Schlottmann S., Barber-Rotenberg J.S., Yuan L., Abaan O.D., Chou T.H., Dakshanamurthy S., Brown M.L. (2009). A small molecule blocking oncogenic protein EWS-FLI1 interaction with RNA helicase A inhibits growth of Ewing's sarcoma. Nat. Med..

[35] Barber-Rotenberg J.S., Selvanathan S.P., Kong Y., Erkizan H.V., Snyder T.M., Hong S.P., Kobs C.L., South N.L., Summer S., Monroe P.J. (2012). Single enantiomer of YK-4-279 demonstrates specificity in targeting the oncogene EWS-FLI1. Oncotarget.

[36] Hu H.M., Zielinska-Kwiatkowska A., Munro K., Wilcox J., Wu D.Y., Yang L., Chansky H.A. (2008). EWS/FLI1 suppresses retinoblastoma protein function and senescence in Ewing's sarcoma cells. J. Orthop. Res..

[37] Potikyan G., France K.A., Carlson M.R., Dong J., Nelson S.F., Denny C.T. (2008). Genetically defined EWS/FLI1 model system suggests mesenchymal origin of Ewing's family tumors. Lab. Invest..

[38] Uren A., Tcherkasskaya O., Toretsky J.A. (2004). Recombinant EWS-FLI1 oncoprotein activates transcription. Biochemistry.

[39] Yon C., Teramoto T., Mueller N., Phelan J., Ganesh V.K., Murthy K.H., Padmanabhan R. (2005). Modulation of the nucleoside triphosphatase/RNA helicase and 5′-RNA triphosphatase activities of Dengue virus type 2 nonstructural protein 3 (NS3) by interaction with NS5, the RNA-dependent RNA polymerase. J. Biol. Chem..

[40] Defenbaugh D.A., Johnson M., Chen R., Zheng Y.Y., Casey J.L. (2009). Hepatitis delta antigen requires a minimum length of the hepatitis delta virus unbranched rod RNA structure for binding. J. Virol..

[41] Griffin B.L., Chasovskikh S., Dritschilo A., Casey J.L. (2014). Hepatitis delta antigen requires a flexible quasi-double-stranded RNA structure to bind and condense hepatitis delta virus RNA in a ribonucleoprotein complex. J. Virol..

[42] Chasovskikh S., Dimtchev A., Smulson M., Dritschilo A. (2005). DNA transitions induced by binding of PARP-1 to cruciform structures in supercoiled plasmids. Cytometry A.

[43] Pietrasanta L.I., Thrower D., Hsieh W., Rao S., Stemmann O., Lechner J., Carbon J., Hansma H. (1999). Probing the Saccharomyces cerevisiae centromeric DNA (CEN DNA)-binding factor 3 (CBF3) kinetochore complex by using atomic force microscopy. Proc. Natl. Acad. Sci. U.S.A..

[44] Schneider S.W., Larmer J., Henderson R.M., Oberleithner H. (1998). Molecular weights of individual proteins correlate with molecular volumes measured by atomic force microscopy. Pflugers Arch..

[45] Roy A., Kucukural A., Zhang Y. (2010). I-TASSER: a unified platform for automated protein structure and function prediction. Nat. Protoc..

[46] Kiefer F., Arnold K., Kunzli M., Bordoli L., Schwede T. (2009). The SWISS-MODEL Repository and associated resources. Nucleic Acids Res..

[47] Kelley L.A., Sternberg M.J. (2009). Protein structure prediction on the Web: a case study using the Phyre server. Nat. Protoc..

[48] Walbott H., Mouffok S., Capeyrou R., Lebaron S., Humbert O., van Tilbeurgh H., Henry Y., Leulliot N. (2010). Prp43p contains a processive helicase structural architecture with a specific regulatory domain. EMBO J..

[49] He Y., Andersen G.R., Nielsen K.H. (2010). Structural basis for the function of DEAH helicases. EMBO Rep..

[50] Emsley P., Lohkamp B., Scott W.G., Cowtan K. (2010). Features and development of Coot. Acta Crystallogr. D Biol. Crystallogr..

[51] Krissinel E., Henrick K. (2004). Secondary-structure matching (SSM), a new tool for fast protein structure alignment in three dimensions. Acta Crystallogr. D Biol. Crystallogr..

[52] Buttner K., Nehring S., Hopfner K.P. (2007). Structural basis for DNA duplex separation by a superfamily-2 helicase. Nat. Struct. Mol. Biol..

[53] Kim D., Pertea G., Trapnell C., Pimentel H., Kelley R., Salzberg S.L. (2013). TopHat2: accurate alignment of transcriptomes in the presence of insertions, deletions and gene fusions. Genome Biol..

[54] Trapnell C., Hendrickson D.G., Sauvageau M., Goff L., Rinn J.L., Pachter L. (2013). Differential analysis of gene regulation at transcript resolution with RNA-seq. Nat. Biotechnol..

[55] Johnson N.L., Kotz S., Kemp A. W. (1992). Univariate Discrete Distributions.

[56] Decker K.F., Zheng D., He Y., Bowman T., Edwards J.R., Jia L. (2012). Persistent androgen receptor-mediated transcription in castration-resistant prostate cancer under androgen-deprived conditions. Nucleic Acids Res..

[57] Nishi R., Sakai W., Tone D., Hanaoka F., Sugasawa K. (2013). Structure-function analysis of the EF-hand protein centrin-2 for its intracellular localization and nucleotide excision repair. Nucleic Acids Res..

[58] Barron V.A., Zhu H., Hinman M.N., Ladd A.N., Lou H. (2010). The neurofibromatosis type I pre-mRNA is a novel target of CELF protein-mediated splicing regulation. Nucleic Acids Res..

[59] Farooqui A.A. (1980). Purification of enzymes by heparin-sepharose affinity chromatography. J. Chromatogr..

[60] Scopes R.K. (1993). Protein isolation at the Centre for Protein and Enzyme Technology at La Trobe University. Australas. Biotechnol..

[61] Claude A., Arenas J., Hurwitz J. (1991). The isolation and characterization of an RNA helicase from nuclear extracts of HeLa cells. J. Biol. Chem..

[62] Xing L., Liang C., Kleiman L. (2011). Coordinate roles of Gag and RNA helicase A in promoting the annealing of formula to HIV-1 RNA. J. Virol..

[63] Wang L., Brown S.J. (2006). BindN: a web-based tool for efficient prediction of DNA and RNA binding sites in amino acid sequences. Nucleic Acids Res..

[64] Terribilini M., Sander J.D., Lee J.H., Zaback P., Jernigan R.L., Honavar V., Dobbs D. (2007). RNABindR: a server for analyzing and predicting RNA-binding sites in proteins. Nucleic Acids Res..

[65] Agostini F., Zanzoni A., Klus P., Marchese D., Cirillo D., Tartaglia G.G. (2013). catRAPID omics: a web server for large-scale prediction of protein-RNA interactions. Bioinformatics.

[66] Hong S.H., Youbi S.E., Hong S.P., Kallakury B., Monroe P., Erkizan H.V., Barber-Rotenberg J.S., Houghton P., Uren A., Toretsky J.A. (2014). Pharmacokinetic modeling optimizes inhibition of the ‘undruggable’ EWS-FLI1 transcription factor in Ewing Sarcoma. Oncotarget.

[67] Eden E., Navon R., Steinfeld I., Lipson D., Yakhini Z. (2009). GOrilla: a tool for discovery and visualization of enriched GO terms in ranked gene lists. BMC Bioinformatics.

[68] Eden E., Lipson D., Yogev S., Yakhini Z. (2007). Discovering motifs in ranked lists of DNA sequences. PLoS Comput. Biol..

[69] Finn R.D., Mistry J., Tate J., Coggill P., Heger A., Pollington J.E., Gavin O.L., Gunasekaran P., Ceric G., Forslund K. (2010). The Pfam protein families database. Nucleic Acids Res..

[70] Koh H.R., Xing L., Kleiman L., Myong S. (2014). Repetitive RNA unwinding by RNA helicase A facilitates RNA annealing. Nucleic Acids Res..

[71] Xing L., Zhao X., Niu M., Kleiman L. (2014). Helicase associated 2 domain is essential for helicase activity of RNA helicase A. Biochim. Biophys. Acta..

[72] Morra R., Smith E.R., Yokoyama R., Lucchesi J.C. (2008). The MLE subunit of the Drosophila MSL complex uses its ATPase activity for dosage compensation and its helicase activity for targeting. Mol. Cell. Biol..

[73] Lee C.G., Chang K.A., Kuroda M.I., Hurwitz J. (1997). The NTPase/helicase activities of Drosophila maleless, an essential factor in dosage compensation. EMBO J..

[74] Delattre O., Zucman J., Plougastel B., Desmaze C., Melot T., Peter M., Kovar H., Joubert I., de Jong P., Rouleau G. (1992). Gene fusion with an ETS DNA-binding domain caused by chromosome translocation in human tumours. Nature.

[75] Dubnau J., Struhl G. (1996). RNA recognition and translational regulation by a homeodomain protein. Nature.

[76] Rivera-Pomar R., Niessing D., Schmidt-Ott U., Gehring W.J., Jackle H. (1996). RNA binding and translational suppression by bicoid. Nature.

[77] Sharrocks A.D. (2001). The ETS-domain transcription factor family. Nat. Rev. Mol. Cell Biol..

[78] Mozaffari-Jovin S., Wandersleben T., Santos K.F., Will C.L., Luhrmann R., Wahl M.C. (2013). Inhibition of RNA helicase Brr2 by the C-terminal tail of the spliceosomal protein Prp8. Science.

[79] Chakrabarti S., Jayachandran U., Bonneau F., Fiorini F., Basquin C., Domcke S., Le Hir H., Conti E. (2011). Molecular mechanisms for the RNA-dependent ATPase activity of Upf1 and its regulation by Upf2. Mol. Cell.

