# Supplementary material for: RNA helicase A activity is inhibited by oncogenic transcription factor EWS-FLI1
Source: Nucleic Acids Res. 2015 Jan 6;43(2):1069–80. doi: 10.1093/nar/gku1328 (PMC4333382; doi:10.1093/nar/gku1328)
Supplement: SUPPLEMENTARY DATA [file supp_43_2_1069__index.html]

RNA helicase A activity is inhibited by oncogenic transcription factor EWS-FLI1 — SUPPLEMENTARY DATA 

# RNA helicase A activity is inhibited by oncogenic transcription factor EWS-FLI1

## SUPPLEMENTARY DATA

**Files in this Data Supplement:**

- SUPPLEMENTARY DATA
- SUPPLEMENTARY DATA
