# Supplementary material for: RNA helicase A activity is inhibited by oncogenic transcription factor EWS-FLI1
Source: Nucleic Acids Res. 2015 Jan 6;43(2):1069–80. doi: 10.1093/nar/gku1328 (PMC4333382; doi:10.1093/nar/gku1328)
Supplement: SUPPLEMENTARY DATA [file supp_gku1328_nar-02886-r-2014-File008.pdf]

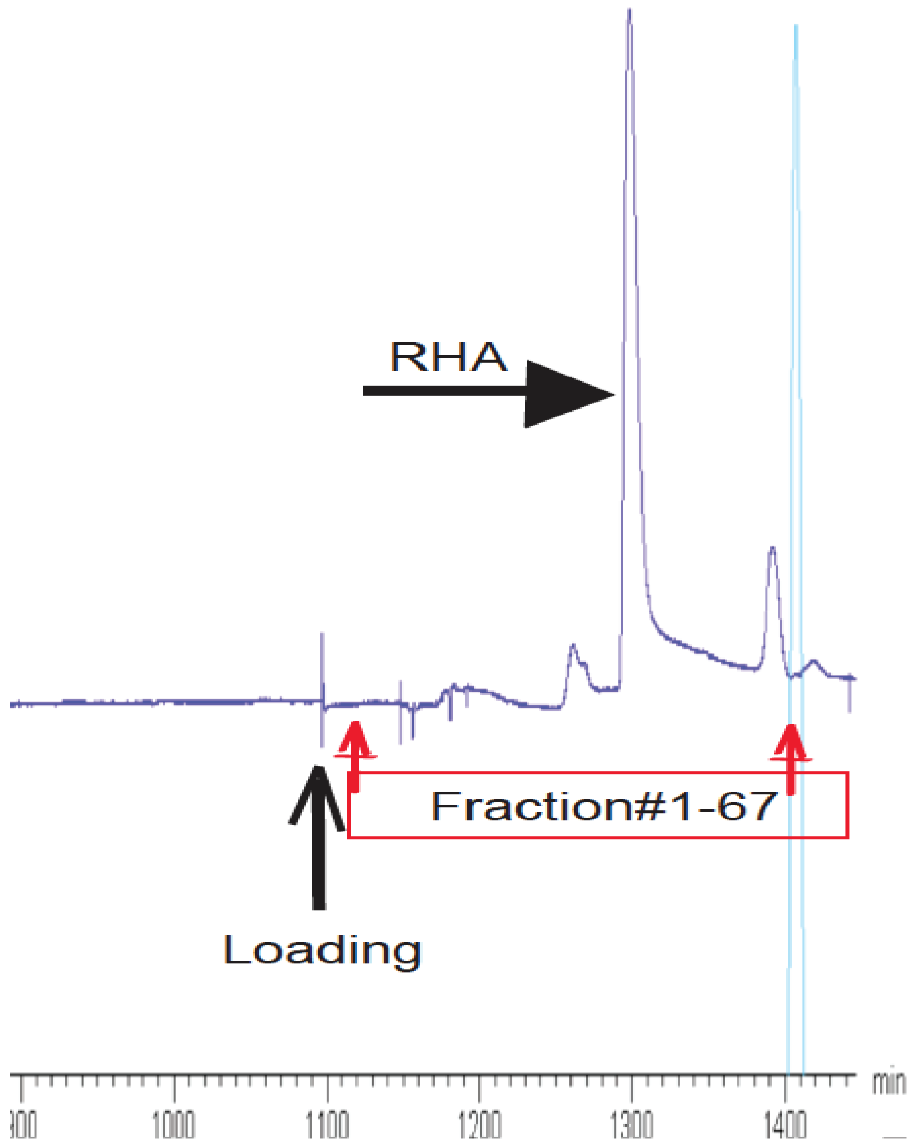

Supplementary Figure 1A. Recombinant RHA is homogenously purified. The purified RHA was loaded to Gel Filtration column containing HiLoad 26/600 Superdex200pg (320 ml) matrix. The sharpness and symetric distribution of the both sides of 280 nm peak demonstrated that purified RHA fractions were homogenously.

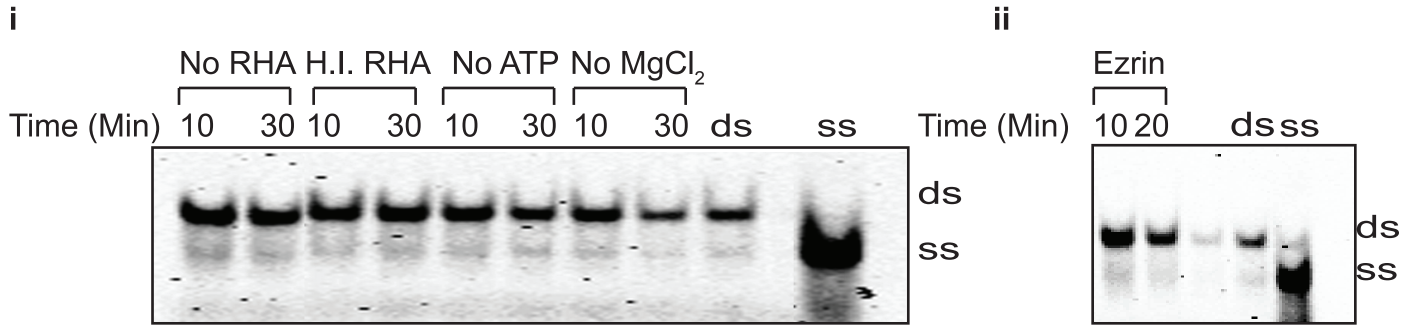

Supplementary Figure 1B: Negative controls for RHA dependent helicase activity. i. Without RHA or with Heat Inactivated (H.I) RHA or without ATP or without $MgCl_2$ strand seperation of dsRNA cannot happen. ii. Non-helicase protein, Ezrin, cannot drive helicase reaction.

Supplementary Figure 1C

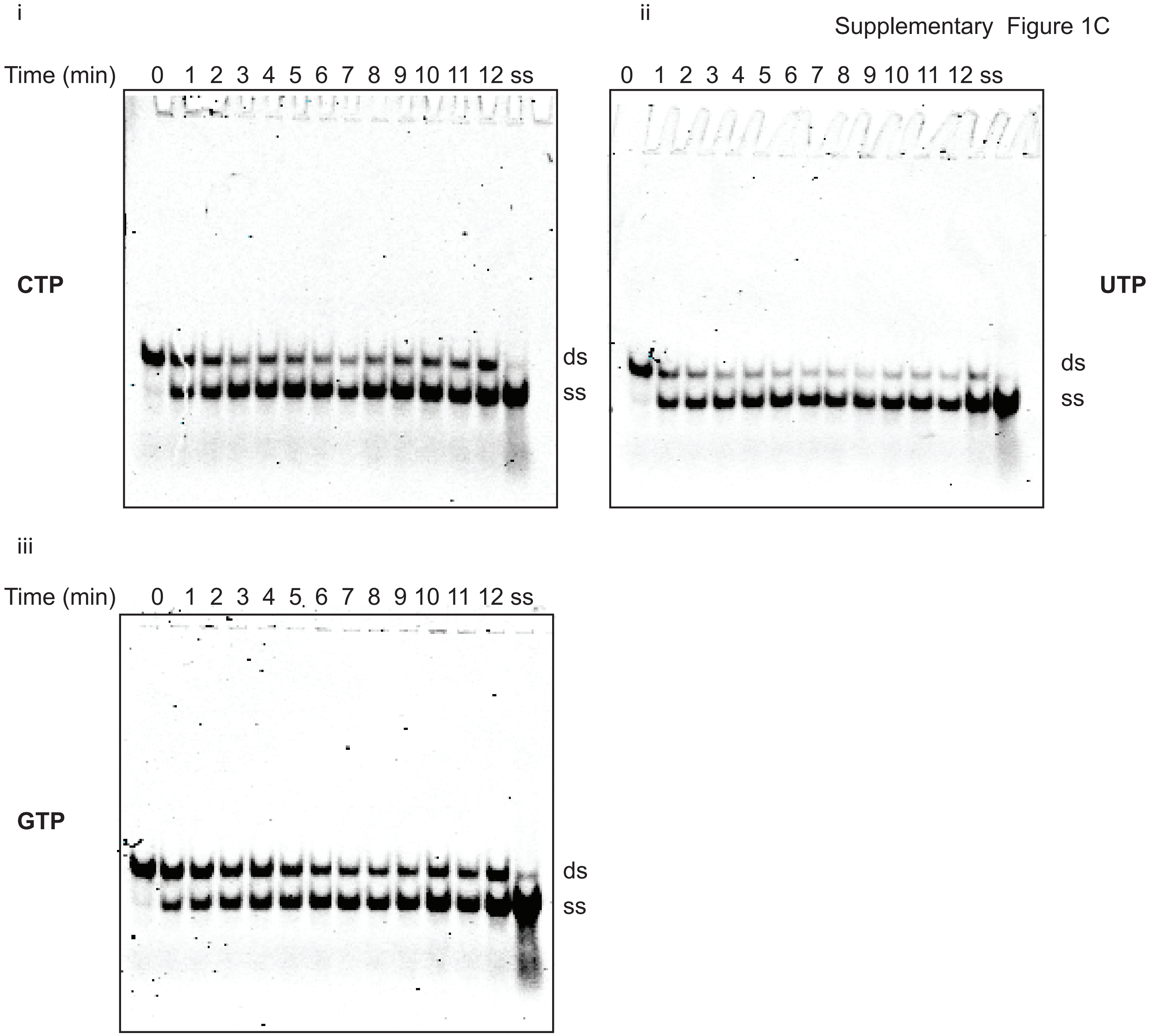

Supplementary Figure 1C. RHA utilizes i.CTP, ii.UTP and iii. GTP in addition to ATP to drive helicase reaction.

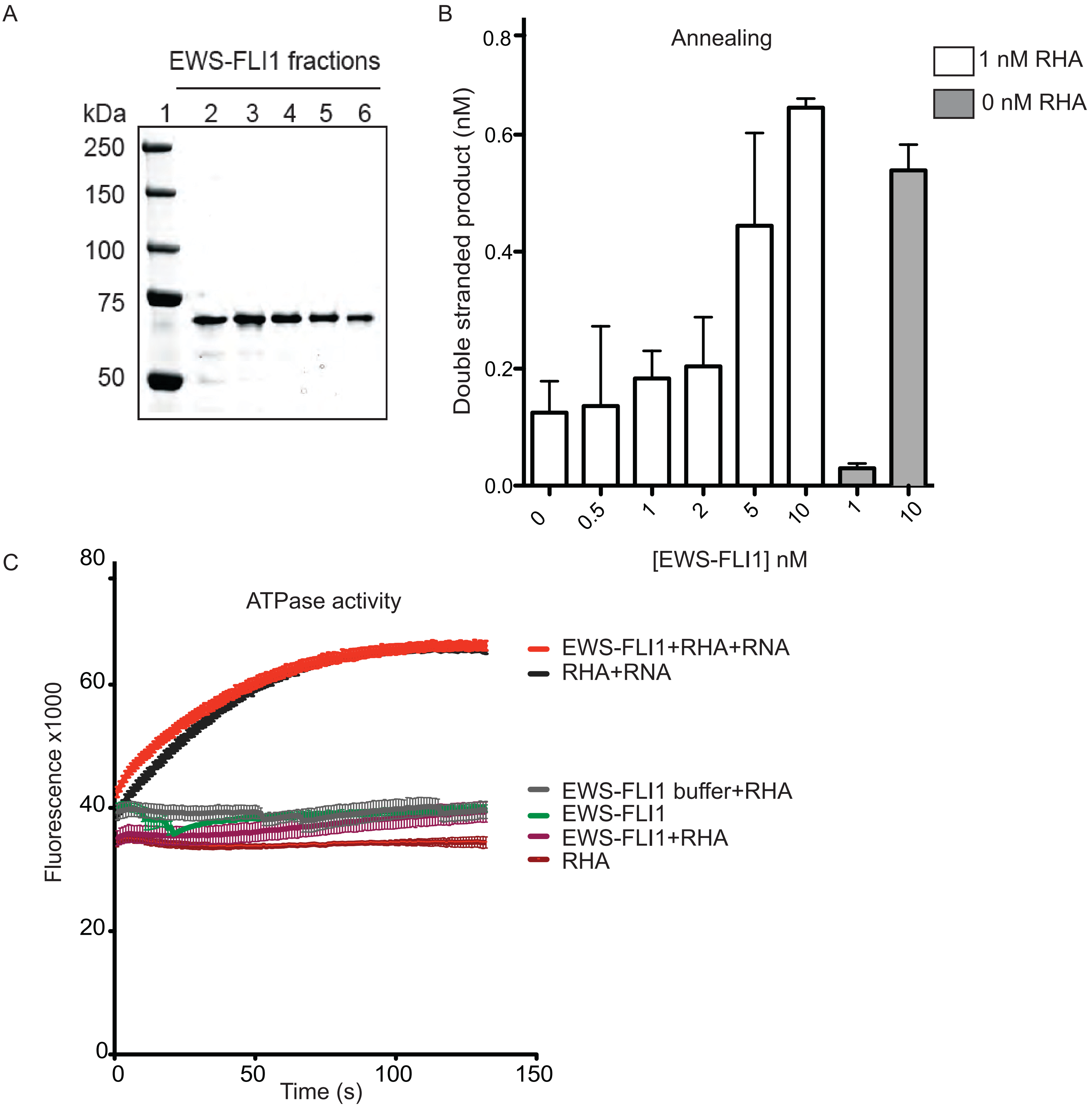

Supplementary Figure S2: A. Coomassie blue stained gel showed the recombinant EWS-FLI1 after purification. B. In two minute reaction, recombinant EWS-FLI1 facilitates the annealing activity of RHA in a dose dependent manner. 10 nM EWS-FLI1 increased the annealing activity of RHA by four fold in 2 minutes. 10 nM EWS-FLI1, alone, facilitated the annealing of ssRNA significantly in 2 minutes. C. EWS-FLI1 did not inhibit the ATPase activity of RHA. A fluorescent substrate was used to measure the ATPase activity of 2 nM RHA and 2 nM RNA (black line) and 2 nM RHA with 2 nM EWS-FLI1 and 2 nM RNA (red line). The reactions without RNA, such as RHA (cayenne line), EWS-FLI1 and RHA (maroon line), EWS-FLI1 alone (green line) and EWS-FLI1 buffer with RHA (gray line) did not have ATPase activity.

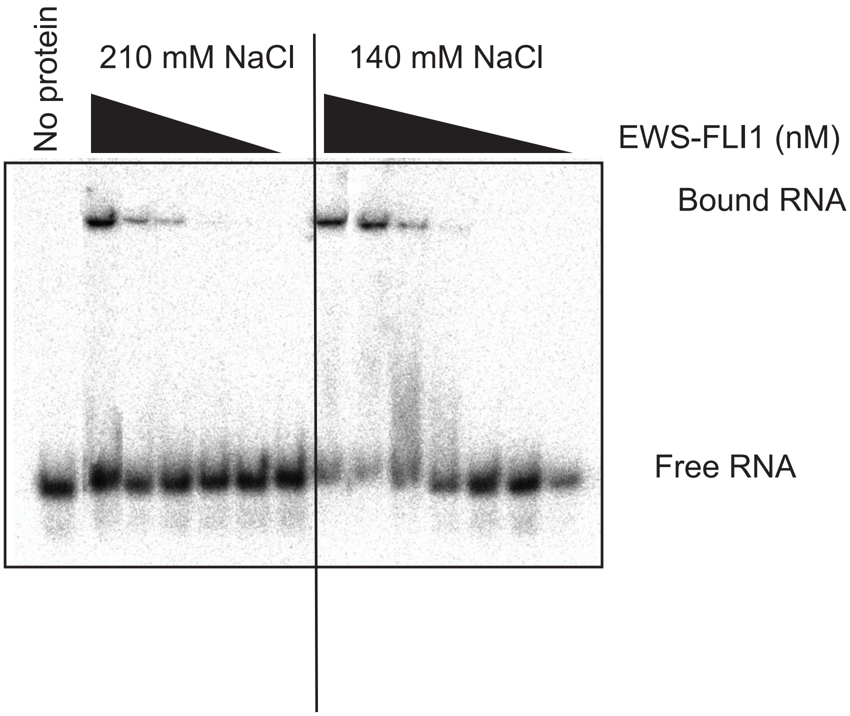

Supplementary Figure 3: Increased salt decreased RNA binding affinity of EWS-FLI1. The RNA binding of EWS-FLI1 was measured in the 140 and 210 nM NaCl containing binding reactions. EWS-FLI1 protein concentration ranged from 0.1 to 312 nM by 5 fold. Free and protein bound RNA was resolved by 6% native PAGE.

catRAPID http://service.tartaglialab.com/ Supplementary Figure 4A

1 MASTDYSTYSQAAAQQGYSAYTAQPTQGYAQTTQAYGQQSYGTYGQPTDVSYTQAQTTTY 60

61 GQTAYATSYGQPPTGYTTPTAPQAYSQPVQGYGTGAYDTTTATVTTTQASYAAQSAYGTQ 120
5

121 PAYPAYGQQPAATAPTRPQDGNKPTETSQPQSSTGGYNQPSLGYGQSNYSYPQVPGSYPM 180
5

181 QPVTAPPSYPPTSYSSTQPTSYDQSSYSQQNTYGQPSSYGQQSSYGQQSSYGQQPPTSYP 240
6

241 PTGSYSQAPSQYSQQSSSYGQQPSYDSVRRGAWGNNMNSGLNKSPPLGGAQTISKNTEQR 300
6 4

301 PQPDPYQILGPTSSRLANPGSGQIQLWQFLLELLSDSANASCITW**EGTNGEFKMTDPDEVARR** 363
4 2 **1**

364 **WGERKSKPNMNYDKLSRALRYYYDKNIMTKVHGKR**YAYKFDFHGIAQALQPHPTESSMYKY 424
2 **1** 3

425 PSDISYMPSYHAHQQKVNFVPPHPSSMPVTSSSFFGAASQYWTSPTGGIYPNPNVPRHPNT 485
3

486 HVPSHLGSYY 495

einstein.cs.iastate.edu/RNABindR **SVM based prediction** Supplementary Figure 4B

MASTDYSTYSQAAAQQGYSAYTAQPTQGYAQTTQAYGQQSYGTYGQPTDVSYTQAQTTTY
0 0 0 0 0 0 0 0 0 0 0 0 0 0 0 0 0 0 0 0 0 0 0 0 0 0 0 0 0 0 0 0 0 0 0 0 0 0 0 0 0 0 0 0 0 0 0 0 0 0 0 0 0 0 0 0 0 0 0 0

GQTAYATSYGQPPTGYTTPTAPQAYSQPVQGYGTGAYDTTTATVTTTQASYAAQSAYGTQ
0 0 0 0 0 0 0 0 0 0 0 0 0 0 0 0 0 0 0 0 0 0 0 0 0 0 0 0 0 0 0 0 0 0 0 0 0 0 0 0 0 0 0 0 0 0 0 0 0 0 0 0 0 0 0 0 0 0 0 0

PAYPAYGQQPAATAPTRPQDGNKPTETSQPQSSTGGYNQPSLGYGQSNYSYPQVPGSYPM
0 0 0 0 0 0 0 0 0 0 0 0 0 0 0 0 0 0 0 0 0 0 0 0 0 0 0 0 0 0 0 0 0 0 0 0 0 0 0 0 0 0 0 0 0 0 0 0 0 0 0 0 0 0 0 0 0 0 0 0

QPVTAPPSYPPTSYSSTQPTSYDQSSYSQQNTYGQPSSYGQQSSYGQQSSYGQQPPTSYP
0 0 0 0 0 0 0 0 0 0 0 0 0 0 0 0 0 0 0 0 0 0 0 0 0 0 0 0 0 0 0 0 0 0 0 0 0 0 0 0 0 0 0 0 0 0 0 0 0 0 0 0 0 0 0 0 0 0 0 0

PTGSYSQAPSQYSQQSSSYGQQPSYDSVRRGAWGNNMNSGLNKSPPLGGAQTISKNTEQR
0 0 0 0 0 0 0 0 0 0 0 0 0 0 0 0 0 0 0 0 0 0 0 0 0 0 0 0 0 0 0 0 0 0 0 0 0 0 0 0 0 0 0 0 0 0 0 0 0 0 0 0 0 0 0 0 0 0 0 0

PQPDPYQILGPTSSRLANPGSGQIQLWQFLLELLSDSANASCITWEGTNGEFKMTDPDEV
0 0 0 0 0 0 0 0 0 0 0 0 0 0 0 0 0 0 0 0 0 0 0 0 0 0 0 0 0 0 0 0 0 0 0 0 0 0 0 0 0 0 0 0 0 0 0 0 0 0 0 0 0 0 0 0 0 0

ARR**WGERKSKPNMNYD**K**LSRAL**R**Y**YYD**K**NI**MTKVHGKR**YA**Y**KFDFHGIAQ**A**L**Q**PHPTESS
0 0 0 **1 1 1 1 1 1 1 1 1 1 1 1 1** 0 **1 1 1 1 1** 0 **1** 0 0 0 **1** 0 0 **1 1 1 1 1 1 1 1** 0 0 **1** 0 0 0 0 0 0 0 0 0 **1** 0 **1** 0 0 0 0 0 0 0 0

MYKYPSDISYMPSYHAHQQKVNFVPPHPSSMPVTSSSFFGAASQYWTSPTGGIYPNPNVP
1 1 1 1 0 0 0 0 0 0 0 0 0 0 0 0 0 0 0 0 0 0 0 0 0 0 0 0 0 0 0 0 0 0 0 0 0 0 0 0 0 0 0 0 0 0 0 0 0 0 0 0 0 0 0 0 0 0 0 0

RHPNTHVP**SH**LG**SY**Y
0 0 0 0 0 0 0 0 **1 1** 0 0 **1 1** 0

Supplementary Figure 4C

www.//bioinfo.ggc.org/cgi-bin/bindn/bindn.pl

```
Sequence:   MASTDYSTYSQAAAQQGYSAYTAQPTQGYAQTTQAYGQQSYGTYGQPTDVSYTQAQTTTY
Prediction: --+--++++-+---++-++-++-+-++-+-++++-+-++++-++---++-++++-+++++
Confidence: 665226655273435427636626256283666728256582784325564667276777

Sequence:   GQTAYATSYGQPPTGYTTPTAPQAYSQPVQGYGTGAYDTTTATVTTTQASYAAQSAYGTQ
Prediction: +++-+-++++++++++++++-++-++++-+-+-+--+-+++-+-++++-++--++-+-++
Confidence: 567282678586784866562674755546443722526662746665567436637366

Sequence:   PAYPAYGQQPAATAPTRPQDGNKPTETSQPQSSTGGYNQPSLGYGQSNYSYPQVPGSYPM
Prediction: --+--+--+--++++++++++++++++++++++++++++++-++-++++++-+---++--
Confidence: 337246525244857796887786787897888976765553582878667364235443

Sequence:   QPVTAPPSYPPTSYSSTQPTSYDQSSYSQQNTYGQPSSYGQQSSYGQQSSYGQQPPTSYP
Prediction: +--+-++++++++++++++++++++++++++++++++++++++++++++++-++++++++
Confidence: 447545478678788989677758677797778587789698778698788298668886

Sequence:   PTGSYSQAPSQYSQQSSSYGQQPSYDSVRRGAWGNNMNSGLNKSPPLGGAQTISKNTEQR
Prediction: +++++++++++++++++++++++++++-++--+-++-+++-+++---+--++-+++++++
Confidence: 884788847778788778879756755588236264457625852225246726887688

Sequence:   PQPDPYQILGPTSSRLANPGSGQIQLWQFLLELLSDSANASCITWEGTNGEFKMTDPDEV
Prediction: ++---------++++-++-++-+------------------------+--+-+-------
Confidence: 462333575335679258247265297799999362523247233652554563774344

Sequence:   ARRWGERKSKPNMNYDKLSRALRYYYDKNIMTKVHGKRYAYKFDFHGIAQALQPHPTESS
Prediction: -++++++++++++++-+-++--++---+---++-+-+++-++--------------++++
Confidence: 476768987978455456583386238527547452887466878689937624235556

Sequence:   MYKYPSDISYMPSYHAHQQKVNFVPPHPSSMPVTSSSFFGAASQYWTSPTGGIYPNPNVP
Prediction: -++--+--++---++-+--+--------++-+-+++------+-++-+-++--+---+--
Confidence: 266234374553265362358347364367252565253245525525264236322532

Sequence:   RHPNTHVPSHLGSYY
Prediction: +-++++--++--+--
Confidence: 824565625444523
```

Supplementary Figure 4: The RNA binding site prediction results for EWS-FLI1.
A. The prediction result of catRAPID (http://service.tartaglialab.com/), B. The prediction result of RNABindR (einstein.cs.iastate.edu/RNABindR) C. The prediction result of BindN (//bioinfo.ggc.org/cgi-bin/bindn/bindn.pl). The boxed regions in all three prediction results show the ets-binding domain of FLI1 protein.

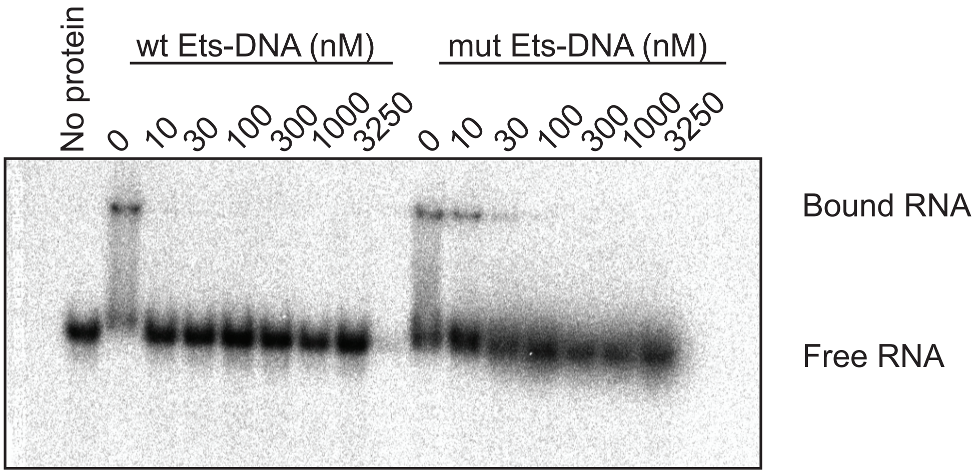

wt Ets-DNA sequence

5’-ATG TAG ACC **GGA A**GT AAC TA-3’
3’-TAC ATC TGG **CCT T**CA TTG AT-5’

mut Ets-DNA sequence

5’-ATG TAG ACC **GCT A**GT AAC TA-3’
3’-TAC ATC TGG **CGA T**CA TTG AT-5’

Supplementary Figure 5: The RNA binding of EWS-FLI1 could be partially through the *ets*-DNA binding domain. Wild- type DNA containing GGAA (bold and underlined) or mutant GCTA sequences (bold and boxed) was included in the RNA binding assays of EWS-FLI1. 30 nM EWS-FLI1 was complexed with varying concentration from 0 to 3250 nM of the ets-DNA before the addition of dsRNA. The protein-RNA complexes were resolved with 6% native PAGE.

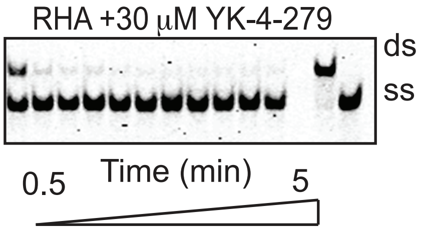

Supplementary Figure 6**:** YK-4-279 does not inhibit the helicase activity of RHA. 30 μM YK-4-279 was added to helicase assay containing 1 nM RHA.

Supplementary Figure 7

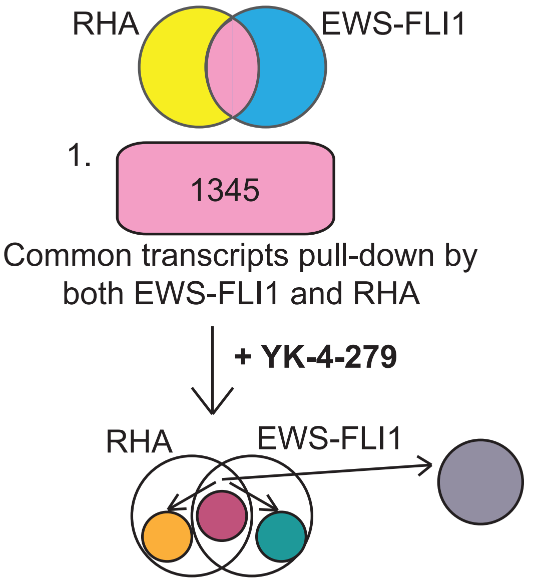

A

1345 by both RHA and EWS-FLI1

GO Term Enrichment	P-value
Cell-cell adhesion	8.0×10^{-6}
Inflammatory response	1.8×10^{-5}
Negative regulation of vasculature development	3.3×10^{-5}
Regulation of vasoconstriction	3.3×10^{-5}
Detection of stimulus involved in sensory perception	7.4×10^{-5}
Neuronal action potential	1.1×10^{-4}
Humoral immune response	2.0×10^{-4}
Positive regulation of blood circulation	2.0×10^{-4}
Cell-cell signaling	4.4×10^{-4}
Negative regulation of notch signaling	7.8×10^{-4}
Regulation of ion transport	8.0×10^{-4}

B

615 by both RHA and EWS-FLI1

GO Term Enrichment	P-value
Detection of chemical stimulus involved in sensory perception of smell	9.8×10^{-7}
Leukotrine metabolic process	1.0×10^{-5}
Phosphatidylglycerol metabolic process	1.0×10^{-5}
Phosphatidic acid biosynthetic process	7.2×10^{-4}
Positive regulation of antigen processing and presentation	6.0×10^{-4}
Endochondral ossification	3.3×10^{-4}
Icosanoid biosynthetic process	3.2×10^{-4}
Potassium ion transmembrane transport	1.8×10^{-4}

C

306 by EWS-FLI1

GO Term Enrichment	P-value
Cell communication	5.7×10^{-4}
Single organism signaling	4.7×10^{-4}

D

243 by RHA

GO Term Enrichment	P-value
Histone H4K20 demethylation	6.0×10^{-4}
Negative regulation of megakaryocyte differentation	3.3×10^{-4}

E

181 RNA by neither RHA nor EWS-FLI1

GO Term Enrichment	P-value
Regulation of JAK-STAT cascade	8.4×10^{-4}

Supplementary Figure 7: RIP-seq determined overlapping transcripts from either EWS-FLI1 or RHA complexes. A. Gene Ontology term enrichment analysis of RNA found in both RHA and EWS-FLI1 protein complexes. B. The analysis of RNA continued to be overlapping from both RIP after YK-4-279 treatment. C. The GO analysis of RNA only present with EWS-FLI1 RIP after YK-4-279 treatment. D. GO term enrichment of RNA present in RHA-RIP following YK-4-279 treatment. E. GO enrichment analysis of RNA were no longer RIP by either protein after YK-4-279 treatment.

Supplementary Figure 8

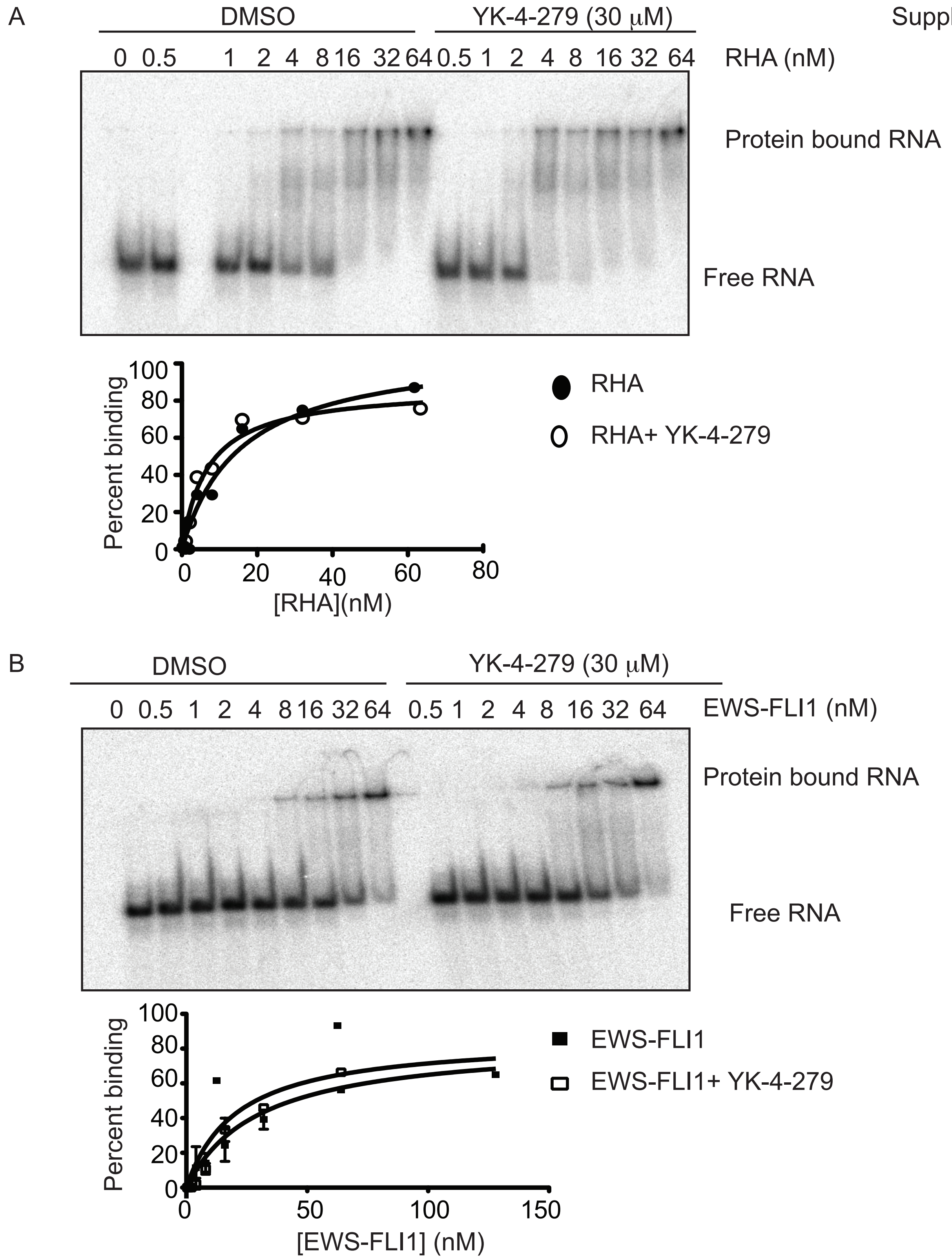

Supplementary Figure 8: YK-4-279 does not affect the RNA binding affinity of RHA or EWS-FLI1.
A. RHA-RNA binding assay was carried out with the varying concentration of RHA in the presence of 30 μM YK-4-279. RNA-protein complexes were resolved in 6% native PAGE. The affinites were calculated by using GraphPad.
B. EWS-FLI1 and RNA binding assay was carried out with the varying concentration of EWS-FLI1 in the presence of 30 μM YK-4-279. RNA-protein complexes were resolved in 6% native PAGE. The affinites were calculated by using GraphPad.

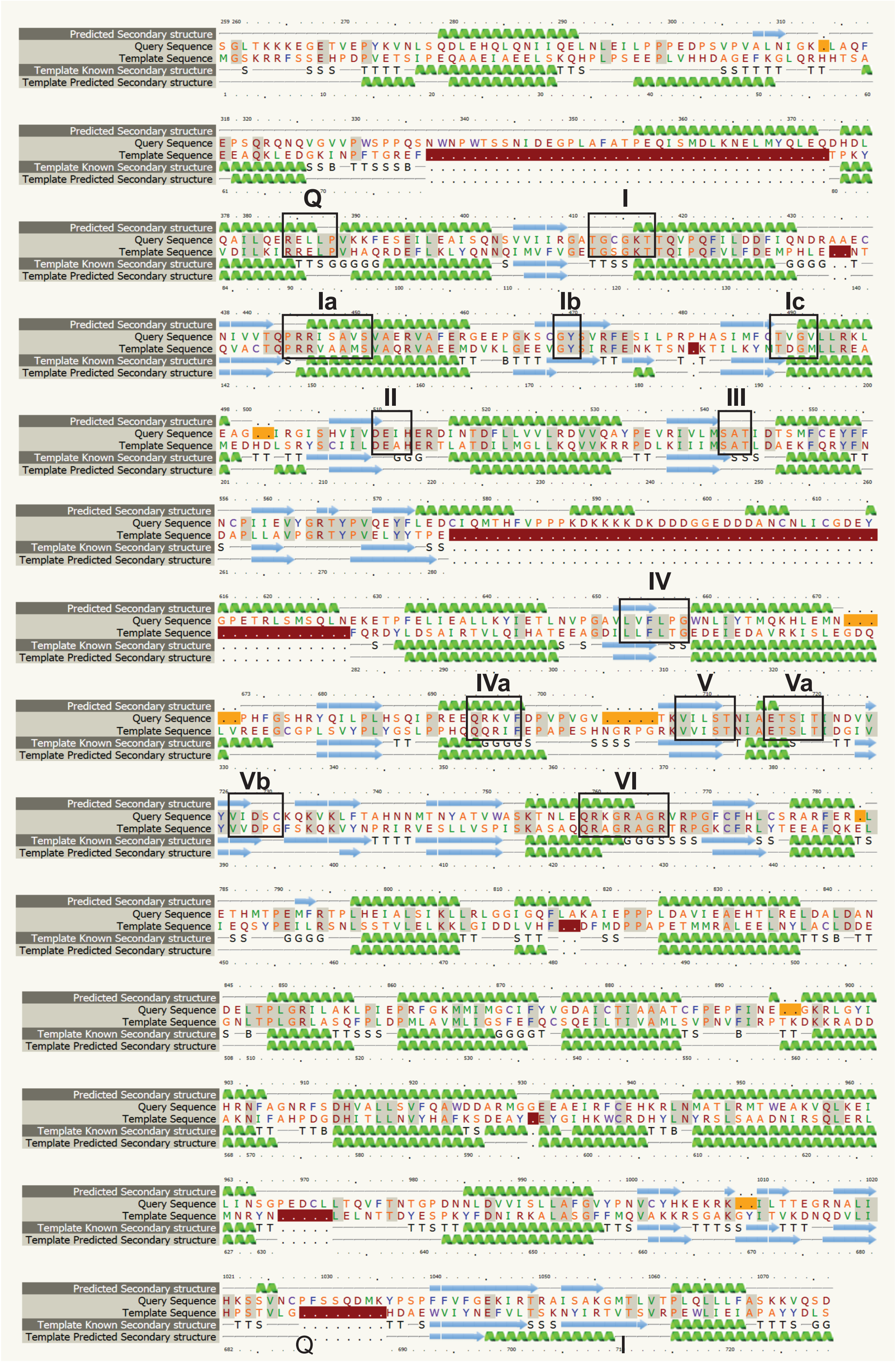

Supplementary Figure S9. Alignment of RHA and S. cerevisiae Prp43p sequences. The alignment generated by PHYRE indicates that the use of S. cerevisiae Prp43p as a template sequence allows not only for the reliable modeling of RHA's overall fold, but also for the modeling of conserved helicase motifs as described by Fairman-Williams et al. (2010). The query sequence belongs to RHA, the template sequence is Prp43p in the alignment.

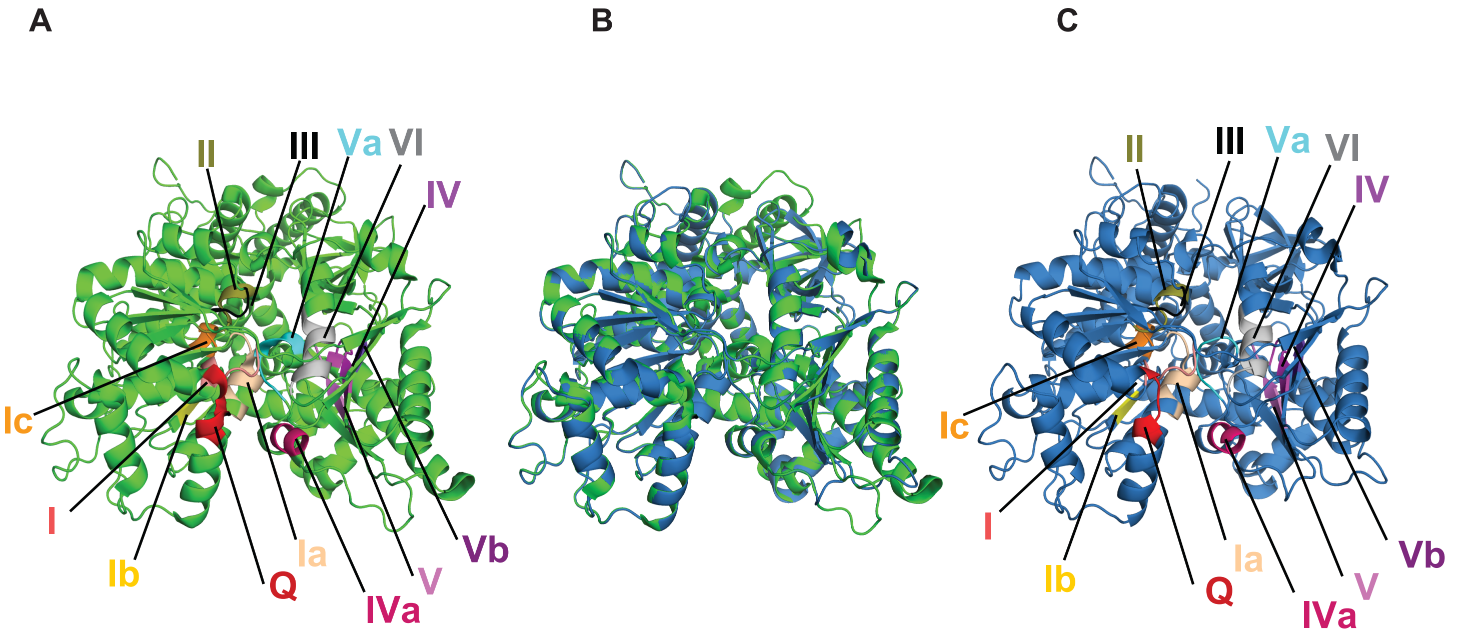

Supplementary figure S10: A. Crystal structure of Prp43p (PDB code: 3KX2) shown in cartoon representation with marked helicase motifs as described by Fairman-Williams et al. (2010). The helicase motifs are those determined for RHA by Fairman-Williams and colleagues.
B. Superposition of the Prp43p crystal structure (green) and modeled core fragment of RHA (blue).
C. The model of RHA with marked helicase motifs.
